# An Amidinohydrolase Provides the Missing Link in the Biosynthesis of Amino Marginolactone Antibiotics

**DOI:** 10.1002/anie.201509300

**Published:** 2015-12-02

**Authors:** Hui Hong, Markiyan Samborskyy, Frederick Lindner, Peter F. Leadlay

**Affiliations:** ^1^ Department of BiochemistryUniversity of Cambridge80 Tennis Court RoadCambridgeCB2 1GAUK; ^2^ Institut für Organische ChemieLeibniz Universität HannoverSchneiderberg 1 B30167HannoverGermany

**Keywords:** amidinohydrolases, biosynthesis, marginolactones, polyketide synthases, streptomyces

## Abstract

Desertomycin A is an aminopolyol polyketide containing a macrolactone ring. We have proposed that desertomycin A and similar compounds (marginolactones) are formed by polyketide synthases primed not with γ‐aminobutanoyl‐CoA but with 4‐guanidinylbutanoyl‐CoA, to avoid facile cyclization of the starter unit. This hypothesis requires that there be a final‐stage de‐amidination of the corresponding guanidino‐substituted natural product, but no enzyme for such a process has been described. We have now identified candidate amidinohydrolase genes within the desertomycin and primycin clusters. Deletion of the putative desertomycin amidinohydrolase gene dstH in Streptomyces macronensis led to the accumulation of desertomycin B, the guanidino form of the antibiotic. Also, purified DstH efficiently catalyzed the in vitro conversion of desertomycin B into the A form. Hence this amidinohydrolase furnishes the missing link in this proposed naturally evolved example of protective‐group chemistry.

Complex polyketides are among the most numerous and structurally diverse bacterial natural products, and they include compounds of outstanding clinical effectiveness, either as antibiotics, immunosuppressants, or antitumor compounds.^1^ They are biosynthesized by polyketide synthase (PKS) multienzymes according to a remarkable assembly‐line paradigm, in which each cycle of polyketide chain extension is accomplished by a different set or module of fatty acid synthase (FAS)‐related enzyme domains.^2^ This provides a direct link between gene sequence and the structure of the chemical product, which means that if a strain is discovered to produce a specific compound, it is now a straightforward procedure to identify the corresponding gene cluster that encodes its biosynthesis. Recent dramatic advances in whole‐genome sequencing also make it possible to make reasonable predictions of the biosynthetic potential of each strain, thereby leading to broad insight into the biogenesis of all major classes of polyketide, and opening the way to “genome mining” for novel compounds.^3^ There is great interest in developing methods of biosynthetic engineering, in partnership with medicinal chemistry, to introduce additional chemical diversity into these molecules.^4^

Desertomycin A (**1 a**, Scheme 1) is a member of the marginolactones, antifungal macrocyclic polyketides substituted with either an amino or a guanidino group and possessing a ring size of 31 carbon atoms or more.^5^ We have recently shown that 4‐guanidinobutanoyl‐CoA derived from l‐arginine provides the starter unit for azalomycin F (**4**, Scheme 1) biosynthesis.^6^ Biosynthesis of the amino‐containing marginolactones has been suggested to follow an analogous pathway from ornithine,^5^, ^7^ but we have previously proposed an alternative hypothesis, in which amino marginolactones are derived from their guanidino‐substituted counterparts in a deprotection^8^, ^9^ step catalyzed by an amidinohydrolase as a late step in biosynthesis. The biosynthetic gene cluster for the aminopolyene ECO‐02301 has been reported to contain a gene for a potential amidinohydrolase enzyme.^10^ We report herein a genome‐based approach to identifying and characterizing amidinohydrolases acting in marginolactone biosynthesis, and we show that the novel amidinohydrolase DstH is indeed necessary and sufficient for the deprotection of desertomycin B^11^ to form desertomycin A. Our results set the stage for a detailed examination of marginolactone biosynthesis, and reveal a new possibility for the designed incorporation of a chemically reactive amino functionality into complex polyketides.

**Scheme 1:**
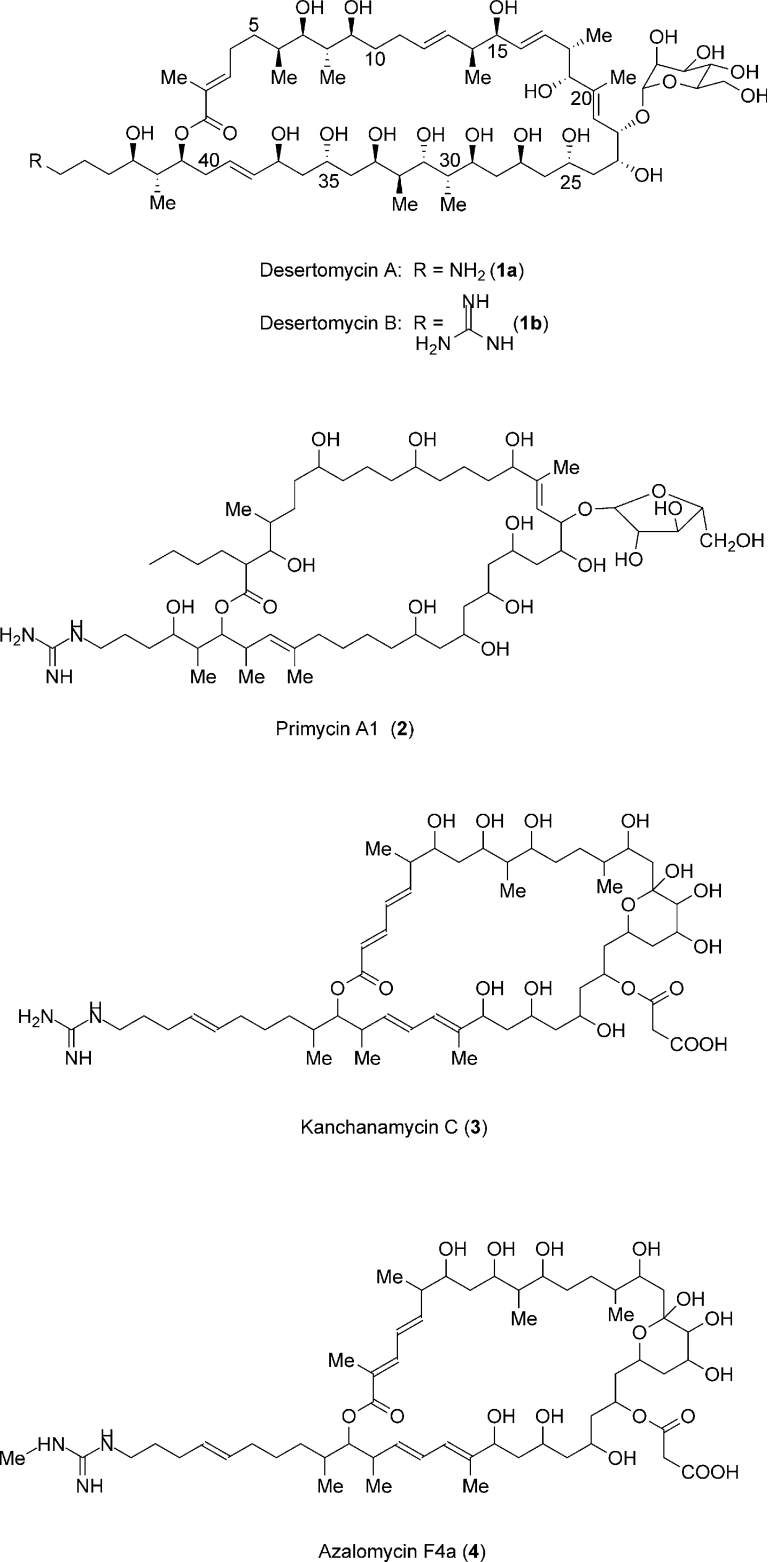
Structures of the antifungal marginolactones desertomycin (**1**), primycin A1 (**2**), kanchanamycin C (**3**), and azalomycin F4a (**4**).

We selected for genome sequence analysis three known desertomycin A producing strains: *Streptomyces olivaceus* Tü4018, which also produces the 36‐membered marginolactone kanchanamycin (**3**, Scheme 1),^12^ *Streptomyces macronensis*,^13^ and *Streptomyces spectabilis*.^14^ We also determined high‐quality whole‐genome sequences for *Saccharomonospora azurea* (*syn. S. caesia*), which produces the 36‐membered arabinosyl marginolactone primycin (**2**, Scheme 1),^15^ and for *Streptomyces violaceusniger* DSM4137^16^ which produces the guanidino marginolactone azalomycin F.^17^ Using a previously characterized arginine oxidase gene^6^ from the *S. violaceusniger* strain as a probe, all six target gene clusters were located within their respective genome sequences. The desertomycin gene cluster in *S. olivaceus* and the primycin gene cluster in *S. azurea* are arranged as shown in Scheme S1 in the Supporting Information. Detailed information about each gene is given in the Supporting Information (Tables S4–S9) for all clusters. The arrangement of enzymatic domains within each modular PKS (Figures S1 and S2 in the Supporting Information), and the predicted configuration of the full‐length polyketide chains (Figure S3), were deduced by using previously validated sequence motifs in each type of domain: acyltransferase (AT),^18^ ketoreductase (KR),^19^ dehydratase (DH),^20^ and enoylreductase (ER)^21^ domains. For primycin and desertomycin,^22^, ^23^ there is (almost) exact correspondence between the enzyme arrangement in each extension module and the chemical structure of the polyketide product. In contrast, for both azalomycin and kanchanamycin PKSs, 20 cycles of chain extension are apparently accomplished by only 19 extension modules. In these gene clusters, the first multienzyme in the PKS assembly line (AzlA1 and KchA1 respectively), which contains the loading acyl carrier protein and the first extension module, appears to carry out both the first and second cycles of chain extension. Such programmed iterative use of a PKS module is unusual but not unprecedented.^24^ Also, the structure of these two marginolactones requires full reduction by extension module 3, yet neither PKS possesses an ER domain in that module. Work is in progress to determine the mechanisms involved. Only for desertomycin has the absolute configuration been experimentally established.^23^, ^25^ Comparison of this with the configuration predicted from the PKS structure (Figures S1–S3) showed exact agreement, except for the configuration at C‐30, which is reversed from that predicted.

The desertomycin‐, primycin‐, and kanchanamycin‐producing strains were all found, upon LC–MS analysis of fermentation extracts, to produce a mixture of guanidino and amino forms (Figures S4–S6 and Tables S10, S12, S13). For desertomycin, the amino form is by far the major form, while for primycin and kanchanamycin, both forms contribute significantly. In contrast, azalomycins were never detected in amino form (Figure S7). As indicated in Scheme S1 and Table S4, an open reading frame (*dst6277*, hereinafter referred to as *dstH*) that is co‐located with the polyketide synthase region of the desertomycin gene cluster in *S. macronensis* is predicted, on the basis of sequence comparisons with public databases, to encode an enzyme in the ureohydrolase superfamily (Figure S8). The ureohydrolase superfamily embraces diverse agmatinases, arginases, guanidinobutyrases, formiminoglutamase, and proclavaminate hydrolase.^26^ The mechanism of these enzymes involves nucleophilic attack on the amidino carbon by a hydroxide ion bridging two divalent metal ions.^26^

It cannot be ruled out that a uniquely essential biosynthetic gene is encoded elsewhere than in the main biosynthetic gene cluster.^27^ However, our interest in the putative amidinohydrolase DstH was strengthened by the finding that it shares 56 % (76 %) amino acid identity (similarity) with PriH, the product of a gene in the primycin gene cluster immediately adjacent to the PKS region (Scheme S1 and Table S8). Sequence alignment of DstH and PriH with authentic ureohydrolases in the Protein Data Bank (PDB) protein structure database (Figure S8 A) revealed that DstH and PriH contain the sequence motifs xGGDH, DAHxD, and SxDxDxxDPxxxP (where x=any amino acid), which are conserved in this enzyme superfamily and are implicated in cation binding and catalysis.^26^ To study the possible role of DstH in desertomycin biosynthesis, we created an in‐frame deletion in the *dstH* gene in *S. macronensis* (Figure S9), as described in the Supporting Information. The resulting mutant strain ΔdstH was grown in liquid culture and analyzed for the production of desertomycins by HPLC–MS. Desertomycin A production was found to be completely abolished in this strain, and instead a new species was detected with a retention time later by 0.8 min, and with *m*/*z* [*M*+H]^+^ 1234.7, which corresponds to the molecular ion for the guanidino form of the antibiotic. This compound, desertomycin B **(1 b**; Figure 1 A) was originally described as a minor fermentation product of *Streptomyces flavofungi*.^11^ Its identity was confirmed by MS/MS and high‐resolution MS analysis (Figure S4 and Tables S10, S11). The level of desertomycin B produced by the mutant is the same as that of desertomycin A in the wild type. This finding strongly supports a mechanism for the production of desertomycin A in which the amidino group is hydrolyzed to liberate the amino form as the last step in the biosynthetic pathway. It also convincingly identifies DstH as the essential and sufficient catalyst for this conversion in vivo. Minor amounts were detected of a putative positional isomer of desertomycin A, labeled 1 a* in Figure 1, and this species was likewise replaced in the mutant strain by 1 b*. To confirm the role of DstH, the gene for this enzyme was cloned and expressed in recombinant *E. coli* and purified to near homogeneity (Figure S10). Desertomycin B was purified from the ΔdstH mutant of *S. macronensis* and tested as a substrate for the enzyme. In the presence of an appropriate divalent metal ion (cobalt, nickel) desertomycin B was efficiently converted into desertomycin A, as judged by LC–MS analysis and comparison with authentic material (Figure 1 B and Figure S11). Kanchanamycin C (**3**; guanidino form) was purified from extracts of *S. olivaceus* Tü4018 and also tested as a substrate for DstH. The *kch* gene cluster of *S. olivaceus* does not contain an analogue of DstH or PriH, so it seemed possible that the DstH enzyme encoded in the desertomycin cluster of the same strain might show crosstalk and be able to perform the final‐stage deprotection of kanchanamycin to the amino form. In accord with this idea, kanchanamycin proved to be a good substrate for DstH with each the added divalent metals tested (Co^2+^, Ni^2+^, Zn^2+^, Mg^2+^, Mn^2+^; Figure 2 B and Figure S13). However, further work will be required to establish the exact identity and status of active‐site metal ions in DstH.

**Figure 1:**
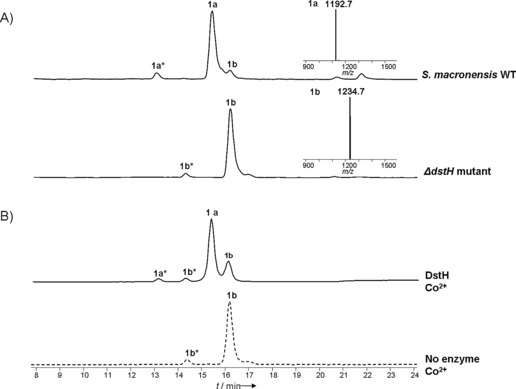
HPLC–MS analysis of desertomycins A (**1 a**) and B (**1 b**). A) LC–ESI‐MS total ion current traces for mycelium methanol extracts from *S. macronensis* wild type and the ΔdstH deletion mutant. In the mutant, production of **1 a** was abolished and replaced by guanidino compound **1 b**. Inserts show mass spectra of [*M*+H]^+^ ions for **1 a** (1192.7) and **1 b** (1234.7). The 13.02 min and 14.27 min peaks labeled as 1 a* and 1 b* have the same MS and MS/MS as **1 a** and **1 b**, respectively, and may represent isomers of **1 a** and **1 b** of altered ring size, although this remains to be established. B) LC–ESI‐MS total ion current traces of DstH‐catalyzed conversion of desertomycin B **1 b** (and putative isomer 1 b*) into desertomycin A **1 a** (and putative isomer 1 a*) when Co^2+^ is present as the activating metal ion.

**Figure 2:**
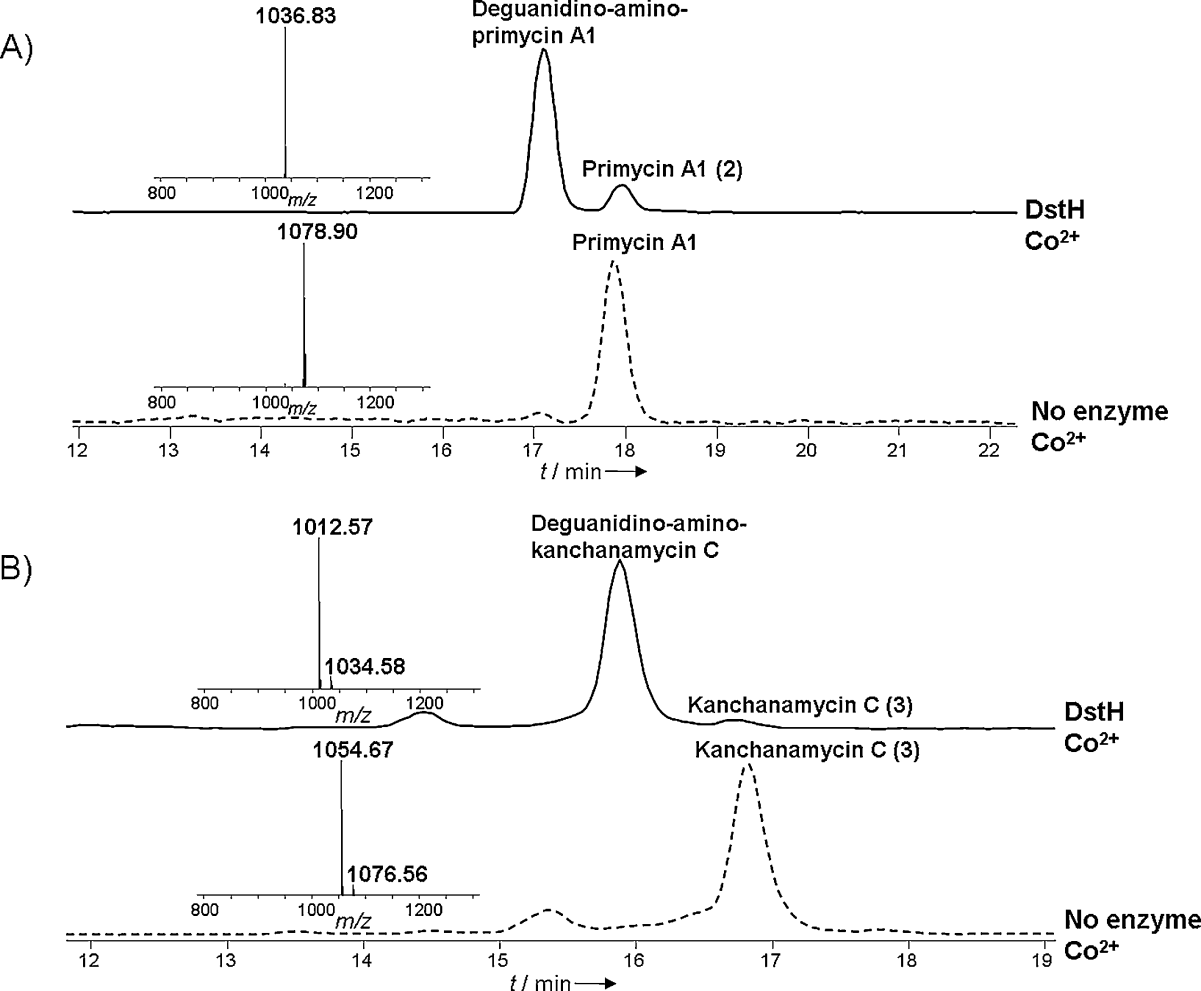
HPLC–MS analysis of in vitro assays with DstH. A) LC–ESI‐MS total ion current traces of DstH‐catalyzed conversion of primycin A1 (**2**) into the amino form. (B) LC–ESI‐MS total ion current traces of DstH‐catalyzed conversion of kanchanamycin C (**3**) into the amino form. For the assays shown, Co^2+^ was the metal ion present. For assays with Ni^2+^, Zn^2+^, Mg^2+^, and Mn^2+^ as the activating metal ion, see Figures S12, S13.

Azalomycin is almost identical to kanchanamycin, except that the major form of azalomycin (**4**, azalomycin F4a) produced by *S. violaceusniger* DSM4137 is methylated on the guanidino group. Azalomycin F4a was not a substrate for purified DstH (Figure S14), thus implying that methylation prevents unmasking of the primary amino group. Given the high sequence identity between DstH and PriH, we predicted that DstH would also act on primycins.15b Primycin A1 (**2**) was purified from *S. caesia* and incubated with DstH in the presence of Co^2+^ or Ni^2+^, where it gave almost complete conversion of guanidino primycins into the corresponding amino forms (Figure 2 A and Figure S12). Therefore DstH amidinohydrolase is flexible enough to accept different macrocyclic polyketides as substrates. This in turn suggests the possibility that, if the guanidinobutanoate starter unit were in the future successfully grafted onto a different macrocyclic polyketide by biosynthetic engineering, DstH or another natural amidinohydrolase could be used in a mild enzymatic deprotection step to expose the primary amino function for selective chemical derivatization.

It is instructive to compare the marginolactone pathway proposed herein with butirosin^8^ and vicenistatin^9^ biosynthesis, where a protective‐group strategy has also been proposed to prevent thioester intermediates being intercepted by intramolecular nucleophilic attack from a primary amine (Scheme 2). It might be argued that, for example, non‐ribosomal peptide synthetases (NRPSs) recruit unprotected amino acids and yet use thioester chemistry,^28^ and modular polyketide synthases are also known that recruit free aromatic^29^ or aliphatic^30^ amino‐substituted building blocks. However, the three cases in Scheme 1 are distinguished from these other examples by the fact that cyclization of specific amino‐substituted thioester intermediates would proceed through the chemically facile formation of either a 5‐ or a 6‐membered ring. For butirosin (Scheme 2 A), γ‐aminobutanoyl‐BtrI is the vulnerable acyl‐ACP intermediate, and for desertomycin (Scheme 2 C), the same hypothetical unprotected acyl‐ACP starter unit would initiate polyketide assembly. For vicenilactam, the aglycone core of vicenistatin, the PKS‐bound thioester intermediate after the first cycle of chain extension (Scheme 2 B) would similarly favor cyclization if it were not protected by the addition of an N‐terminyl l‐Ala unit. In fact, intermediates attached to peptidyl carrier protein (PCP) domains on NRPSs are well known to suffer side reactions when cyclization is sterically favored, especially in the formation of cyclodipeptides.^31^ Ornithinyl‐PCP thioesters are especially liable to this side reaction, presumably because cyclization occurs through attack of a primary amine on a thioester via a 6‐membered transition state to form 3‐amino‐2‐piperidone. The formation on the gramicidin S synthetase NRPS of both cyclo‐ornithine^32^ and cyclo‐ornithinyl peptides^33^ has been demonstrated. Spencer and colleagues in their studies on butirosin reported instability of 4‐aminobutanoyl‐CoA in solution.^8^ In contrast, 4‐guanidylbutanoyl‐CoA and 4‐guanidinylbutanoyl‐ACP are both stable in neutral aqueous buffers at room temperature.^6^ It would appear that protective‐group chemistry in biosynthetic pathways only evolves where it is most needed.

**Scheme 2:**
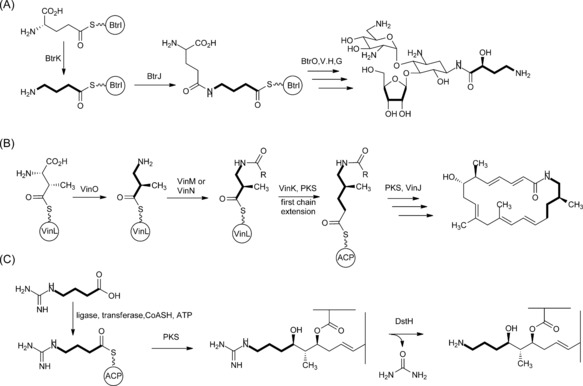
Examples of a protective‐group strategy in the biosynthesis of the natural products butirosin (A); vicenilactam (B), the aglycone core of vicenistatin; and desertomycin (C). Each pathway has at least one intermediate (relevant portion shown in bold) that, unless protected, would be vulnerable to facile cyclization via a five‐ or six‐membered cyclic transition state.

## Supporting information

As a service to our authors and readers, this journal provides supporting information supplied by the authors. Such materials are peer reviewed and may be re‐organized for online delivery, but are not copy‐edited or typeset. Technical support issues arising from supporting information (other than missing files) should be addressed to the authors.

Supplementary
