# Supplementary material for: An Amidinohydrolase Provides the Missing Link in the Biosynthesis of Amino Marginolactone Antibiotics
Source: Angew Chem Int Ed Engl. 2015 Dec 2;55(3):1118–23. doi: 10.1002/anie.201509300 (PMC4737276; doi:10.1002/anie.201509300)
Supplement: Supplementary file 1 — Supplementary [file ANIE-55-1118-s001.pdf]

Angewandte Chemie
Eine Zeitschrift der Gesellschaft Deutscher Chemiker

Supporting Information

An Amidinohydrolase Provides the Missing Link in the Biosynthesis of Amino Marginolactone Antibiotics

Hui Hong, Markiyan Samborskyy, Frederick Lindner, and Peter F. Leadlay**

anie_201509300_sm_miscellaneous_information.pdf

Supporting Information

Table of Contents

1. Supplementary Methods…………………………………………………………...3

1.1. Bacterial strains and culture conditions..3

1.2. Materials, DNA isolation and manipulation...3

1.3. Metabolite analysis and compound isolation...3

1.4. Gene knock-out in *Streptomyces macronensis*...5

1.5. Protein expression and purification..6

1.6. *In vitro* activity assays of DstH..6

2. Supplementary Scheme and Figures………………...………………………………7

Scheme S1. Conserved genes in the desertomycin and primycin biosynthetic gene clusters…………..7

Figure S1. Polyketide synthase (PKS) domain organisation of marginolactone biosynthetic gene clusters...8

Figure S2: Sequence alignment of the PKS KR domains...10

Figure S3: Predicted linear structures for azalomycin, kanchanamycin, primycin and desertomycin based on bioinformatic analysis of PKS domains...11

Figure S4. HPLC-MS analysis of desertomycin production in *Streptomyces macronensis*...................12

Figure S5. HPLC-MS analysis of primycins in *Saccharomonospora azurea*.......................................14

Figure S6. HPLC-MS analysis of desertomycins and kanchanamycins in *Streptomyces olivaceus* Tü4018...16

Figure S7. HPLC-MS analysis of azalomycin F4a in *Streptomyces violaceusniger* DSM4137..17

Figure S8A. Sequence alignment of ureohydrolases..18

Figure S8B. Cladogram of amidinohydrolases AMH_A828, AMH_A821, and homologs...................19

Figure S9. In-frame deletion of amidinohydrolase gene *dstH* in *Streptomyces macronensis*................20

Figure S10. SDS-PAGE analysis of DstH...21

Figure S11. HPLC-ESI-MS analysis of in vitro amidinohydrolysis of desertomycin B catalysed by DstH..21

Figure S12. HPLC-ESI-MS analysis of in vitro conversion of primycin A1 catalyzed by DstH.....22
Figure S13. HPLC-ESI-MS analysis of in vitro conversion of kanchanamycin C catalyzed by DstH.....23
Figure S14. HPLC-ESI-MS analysis of in vitro conversion of azalomycin F4a catalyzed by DstH.....24

3. Supplementary Tables.....24

Table S1. Bacterial strains used in this study.....24
Table S2. Plasmids used in this work.....25
Table S3. Oligonucleotide primers used in this work.....25
Table S4. Properties of genes within the desertomycin/oasomycin biosynthetic gene cluster of *Streptomyces macronensis* (NRRL B12566)(A861).....26
Table S5. Properties of genes within the desertomycin/oasomycin biosynthetic gene cluster of *Streptomyces olivaceus* Tü4018.....28
Table S6. Properties of genes within the desertomycin/oasomycin biosynthetic gene cluster of *Streptomyces spectabilis* NRRL B-2494.....30
Table S7. Properties of genes within the kanchanamycin biosynthetic gene cluster of *Streptomyces olivaceus* Tü4018 (A828).....33
Table S8. Properties of genes within the primycin biosynthetic gene cluster of *Saccharomonospora azurea* DSM43044(A821).....34
Table S9. Properties of genes within the azalomycin biosynthetic gene cluster of *Streptomyces violaceusniger* DSM4137 (A819).....37
Table S10. High-resolution MS analysis of desertomycin A and desertomycin B produced from *Streptomyces macronensis*.....38
Table S11. High-resolution MS analysis of desertomycin B produced from a *dstH*-deletion mutant of *Streptomyces macronensis*.....39
Table S12. High-resolution MS analysis of desertomycin A and kanchanamycins produced from *Streptomyces olivaceus* Tü4018.....39
Table S13. High-resolution MS analysis of primycins produced from S*accharomonospora azurea* DSM 43044.....39
Table S14. High-resolution MS analysis of azalomycin F4a produced from *Streptomyces violaceusniger* DSM4137.....39

4. Supplementary References.....39

Supplementary Methods

1.1. Bacterial strains and culture conditions

Streptomyces olivaceus Tü4018 (desertomycin and kanchanamycin-producing strain) was the kind gift of Pr. Dr. Wolfgang Wohlleben, University of Tübingen. *Streptomyces macronensis* Dietz sp. nov. UC 8271 (NRRL 12566) and *Streptomyces spectabilis* NRRL B2494 (desertomycin-producing strains) were obtained from the Agricultural Research Service Culture Collection, Peoria, USA. *Saccharomonospora azurea* (syn. *S. caesia*) DSM 43044 (primycin-producing strain) and *Streptomyces violaceusniger* DSM 4137 (azalomycin-producing strain) were obtained from the Leibnitz Institut - DSMZ. All strains were maintained on SFM agar (2% soya flour (AYKASOY), 2% D-mannitol, 2% agar) at 30°C. *E. coli* strains were grown in Luria-Bertani (LB) broth (10% tryptone, 5% yeast extract, 10% NaCl) or agar (10% tryptone, 5% yeast extract, 10% NaCl, 2% agar) at 37°C with appropriate antibiotic selection (kanamycin, at 50 µg ml^{-1}).

1.2. Materials, DNA isolation and manipulation.

Bacterial strains, plasmids and oligonucleotides (Eurofins) used in this work are summarized in Tables S1, S2 and S3 respectively. Restriction endonucleases were purchased from New England Biolabs (NEB). T4 DNA ligase and alkaline phosphatase were purchased from Fermentas. All chemicals were from Sigma-Aldrich. Liquid cultures for isolation of genomic DNA were grown in tryptone soya broth (Difco). DNA isolation and manipulation in *Streptomyces*, and *E. coli* were carried out using standard protocols.[1,2] PCR amplifications were carried out using Phusion® High-Fidelity DNA Polymerase (NEB). *E. coli* BL21(DE3) (Novagen) was used for protein expression.

1.3. Metabolite analysis and compound isolation

For small-scale analysis, *Streptomyces macronensis*, *Saccharomonospora azurea*, and *Streptomyces violaceusniger* DSM 4137 strains were grown in liquid TSBY medium (3% TSB (Tryptic Soy Broth), 10.3% sucrose, 0.5% yeast extract) at 30°C and 150 rpm in a rotary incubator for 2-3 days. *Streptomyces olivaceus* Tü4018 was grown in GYM medium (0.4% glucose, 0.4% yeast extract, 1% malt extract, pH 7.2) for 2-3 days. 1 mL samples of culture broth were centrifuged at 20,000 x *g* for 15 min. The mycelia pellets were then extracted with 1 mL of methanol at 60°C for 2 hours. The mixture was spun down and the clear methanol extract was evaporated to dryness and dissolved in 200 µL of methanol. 10 µL of the extract was analyzed by LC-MS. LC-MS analyses were performed on a HPLC (Agilent Technologies 1200 series) coupled to a Thermo Fisher LTQ mass spectrometer fitted with an electrospray ionization (ESI) source. For extracts from *Streptomyces macronensis* and from *Streptomyces olivaceus* Tü4018, a Luna 5µ C18 column (2.0 x 250 mm, Phenomenex) was used, and the samples were eluted using MQ containing 0.1% formic acid (A) and acetonitrile containing 0.1% formic

acid (B) at a flow rate of 0.2 ml min^{-1}. The linear elution gradient for extracts from *Streptomyces macronensis* was 25% to 50% B over 20 min, 50% to 100% B over 9 min. The elution gradient for extracts from *Streptomyces olivaceus* Tü4018 was 25% to 50% B over 15 min, 50% to 75% B over 30 min, 75% to 100% B over 4 min. For extracts from *Saccharomonospora azurea* and from *Streptomyces violaceusniger* DSM 4137, a Prodigy 5μ C18 column (4.6 x 250 mm, Phenomenex) was used, and the samples were eluted using MQ containing 20mM ammonium acetate (A) and methanol (B) at a flow rate of 0.7 ml min^{-1}. The elution gradient for both extracts was 60% to 95% B over 30 min. The mass spectrometer was run in positive ionization mode, scanning from *m/z* 200 to 2000 in full scan mode. MS/MS analysis were performed on $[M+H]^+$ ions with a normalized collision energy of 30%. High-resolution mass analysis was carried out on Thermo Fisher Orbitrap mass spectrometer with resolution set up at 60 K.

For desertomycin B production and isolation, six 250 ml Erlenmeyer flasks with spirals, containing 50 ml TSBY medium, were inoculated with 1 ml 2-day TSBY seed culture of *S. macronensis dstH*-deletion mutant, and incubated at 30 °C, 200 rpm. After 3 days, the broth was centrifuged at 9,000 rpm for 20 min. The pellet was resuspended in methanol and incubated at 60 °C for 2 h. The methanol extract of mycelia pellets was evaporated to dryness under reduced pressure with the rotary evaporator. The residue was dissolved in methanol, and desertomycin B was purified from a preparative HPLC (Agilent 1200) fitted with a Luna C18 column (100Å, 21.20 x 250 mm, Phenomenex). Compounds were eluted with MQ containing 0.1% formic acid (A) and MeCN containing 0.1% formic acid (B) with a linear gradient of 5% to 35% B over 10 min, 35% to 65% B over 15 min, 65% to 100% B over 10 min at a flow rate of 20 ml/min. Fractions were collected, and checked by MS analysis. Fractions containing desertomycin B were combined. Acetonitrile was removed under reduced pressure, and sample was lyophilized.

For kanchanamycin C production and isolation, six 1 L Erlenmeyer flasks with spirals, containing 250 ml GYM medium, were inoculated with 2.5 ml 2-day GYM seed culture of *Streptomyces olivaceus* Tü4018 and incubated at 30 °C, 200 rpm. After 6 days, the broth was centrifuged at 9,000 rpm for 20 min. The pellet was resuspended in methanol and incubated at 60 °C for 2 h. The suspension was centrifuged in Falcon tubes at 2,500 rpm for 15 min. The supernatants were combined and filtered into a 1 L round flask. The methanol was removed under reduced pressure with the rotary evaporator to give a yellowish residue. The residue was extracted two times with diethyl ether/water. The diethyl ether was removed with the rotary evaporator. After lyophilisation the residues were dissolved in methanol for purification by preparative HPLC. Compounds were eluted with 5 mM ammonium acetate (A) and methanol (B) with a linear gradient of 60% B to 95 % B over 30 min, at a flow rate of 20 ml/min. Fractions were collected, and checked by MS analysis. Fractions containing kanchanamycin C were combined. After removing the methanol under reduced pressure, sample was lyophilized.

For primycin A1 production and isolation, 1 ml 2-day TSBY seed culture of *S. caesia* was inoculated into 100 ml inoculum medium[3] (3% soya flour, 5% wheat starch, 2% NaCl, 0.75% $CaCO_3$,

0.5% Sunflower oil) in a 500 ml Erlenmeyer flask with spiral at 30 °C, 240 rpm. After 2 days, five 500 ml Erlenmeyer flask, containing 100 ml of fermentation medium (5% soya flour, 5% wheat starch, 2% NaCl, 0.75% $CaCO_3$, 0.6% sunflower oil, 0.4% stearic acid, 0.1% KH_2PO_4) , were inoculated with 10 ml inoculum medium and cultivated for 48 h at 30 °C and 240 rpm. Under this fermentation conditions, primycin A1 became the major component. The cultures were centrifuged at 9,000 rpm for 20 min. The pellet was resuspended in methanol and incubated at 60 °C for 2 h. The methanol extract of mycelia pellets was evaporated to dryness under reduced pressure with the rotary evaporator. The residue was dissolved in MeOH, and was purified by solid phase extraction using ISOLUTE® C18 (EC) SPE columns. Primycin A1 was eluted with 60 % acetonitrile/ 40 % milliQ water. After removing the acetonitrile under reduced pressure, sample was lyophilized.

For azalomycin F4a production and isolation, six 1 L Erlenmeyer flasks with spirals, containing 250 ml TSBY medium, were inoculated with 2.5 ml 2-day TSBY seed culture of *Streptomyces violaceusniger* DSM 4137 and incubated at 30 °C, 200 rpm. After 3 days, the broth was centrifuged at 9,000 rpm for 20 min. The pellet was resuspended in methanol and incubated at 60 °C for 2 h. The methanol extract of mycelia pellets was evaporated to dryness under reduced pressure with the rotary evaporator. The residue was dissolved in MeOH, and separated on a sephadex LH20 column with MeOH/chloroform (1:1). The fractions were checked by MS. Fractions containing azalomycin F4a were combined, and solvents were removed under reduced pressure. The residue was dissolved in MeOH, and further purified by semi-preparative HPLC on a Prodigy C18 column (10 x 250 mm, Phenomenex) with a linear gradient of 45% MeCN, 55% 5 mM ammonium acetate to 56% MeCN, 44% 5 mM ammonium acetate over 35 minutes with a flow rate of 10 ml/min. Fractions containing azalomycin F4a were combined. Acetonitrile was removed under reduced pressure, sample was lyophilized.

1.4. Gene knock-out in *S. macronensis*

The amidinohydrolase gene *dstH* in *S. macronensis* was knocked out by in-frame deletion. To construct the deletion plasmid pYH7-*dstH*, *dstH* upstream and downstream fragments (about 2 kb) were amplified from *S. macronensis* genomic DNA by PCR with primers dstH-up F, dstH-up R and dstH-dn F, dstH-dn R, respectively. The cloning vector pYH7[4] was digested with *NdeI*, treated with shrimp alkaline phosphatase (SAP) and gel purified. To ligate the two fragments into pYH7, the isothermal assembly method was used as described.[5] The mixture was incubated at 50°C for 60 min, and then was used to transform *E. coli* DH10B. The integrity of the plasmid was checked by restriction digestion and sequencing.

The construct was then introduced by conjugation into *S. macronensis*. The donor strain was *E. coli* ET12657/pUZ8002, and conjugation was carried out on 20 ml of SFM plates (2% mannitol, 2% soya flour, 2% agar). After incubating at 30°C for 20 hours, exconjugants were selected with 50 µg ml^{-1} apramycin and 25 µg ml^{-1} nalidixic acid. Single colonies from this plate were transferred to a SFM plate

containing 50 μg ml^{-1} apramycin to double check for antibiotic resistance. Mutant screening was carried out by streaking transformants on SFM agar medium for non-selective growth, then patching single colonies onto both SFM agar and SFM agar containing apramycin (50 μg ml^{-1}) in parallel. Candidate colonies with the correct phenotype (Apr^{S}) were selected for further screening by PCR with a pair of primers dstH-CP1 and dstH-CP2 to identify double cross-over mutants. The PCR fragments from the double cross-over mutants were further verified by sequencing.

1.5. Protein expression and purification

The *dstH* gene was amplified by PCR, using genomic DNA of *Streptomyces olivaceus* Tü4018 as template, and inserted into vector pET28a via *Nde*I and *Hin*dIII restriction sites to yield pET28a-*dstH*. The identity of the plasmid was confirmed by DNA sequencing.

The pET28a-*dstH* was then used to transform *E. coli* BL21(DE3) for protein expression. A single colony was inoculated into 10 mL of LB medium containing 50 μg ml^{-1} kanamycin and grown overnight at 37°C, 250 rpm. An aliquot (1 mL) was retained for preparation of a glycerol stock and the remaining culture was inoculated into 1 L LB medium containing 50 μg ml^{-1} kanamycin and incubated at 37°C, 200 rpm until A_{600} reached 0.6 before addition of 400 μL of 1 M isopropyl-β-D-thiogalactopyranoside (IPTG) and incubation at 22°C overnight to induce protein expression. Cells were harvested by centrifugation at 4,000 rpm for 10 min, resuspended in lysis buffer (20 mM Tris-HCl, pH 7.8, 0.5 M NaCl, 10 mM imidazole) and lysed by sonication. The total lysate was centrifuged at 14,000 rpm for 40 min, and the supernatant was loaded onto a His-Bind column (1 mL bed volume), which had been pre-charged with nickel ions and equilibrated with lysis buffer. The column was washed with 10 column volumes of lysis buffer. Bound proteins were then eluted with a step gradient of increasing imidazole concentration (40, 80, 100, 150, 200, 250 and 500 mM in binding buffer). The protein solutions were concentrated, and further purified by gel filtration on an ÄKTA Explorer FPLC system fitted with a HiLoad 16/60 Superdex 200 Prep Grade column. The mobile phase contained 100 mM potassium phosphate, pH 7.4. Fractions containing protein of the expected size were pooled and concentrated using Amicon Ultra-4 concentrators (Millipore) fitted with a 30 kDa filter. All purification steps were carried out at 4°C. The purity of the protein was examined by 4 - 12% Bis-Tris Gel (Novex) analysis and the concentration of the protein was measured by Bradford assay using bovine serum albumin as a standard.

1.6. *In vitro* activity assays of DstH

Each reaction mixture (25 μl) contained 5 μM purified DstH, 1 mM $CoCl_2$ (or $NiCl_2$, $MnCl_2$, $ZnCl_2$, $MgCl_2$, MQ as no-metal control), in 50 mM Tris-HCl buffer pH 9.0. After incubation at 37°C for 30 min, 0.5 μl of purified desertomycin B (or primycin A1, kanchanamycin C, azalomycin F4a) stock solution (in DMSO) was added to a final concentration of 0.3 mM, and the reaction was allowed

to continue at 37°C for 3 hr. 10 μl of the reaction mixture was taken, mixed with 50 μl methanol, and analyzed by HPLC-MS with a Luna 5μ C18 column (2.0 x 250 mm, Phenomenex) eluting with MQ containing 0.1% formic acid (A) and acetonitrile containing 0.1% formic acid (B) at a flow rate of 0.2 ml min^{-1}. The linear elution gradient for assays when desertomycin B or primycin A1 was used as substrate was 25% to 50% B over 20 min, 50% to 100% B over 9 min. The elution gradient for assays when kanchanamycin C was used as substrate was 25% to 50% B over 9 min, 50% to 72% B over 26 min, 72% to 100% B over 5 min. The elution gradient for assays when azalomycin F4a was used as substrate was 25% to 50% B over 5 min, 50% to 75% B over 20 min, 75% to 100% B over 5 min.

2. Supplementary Scheme and Figures

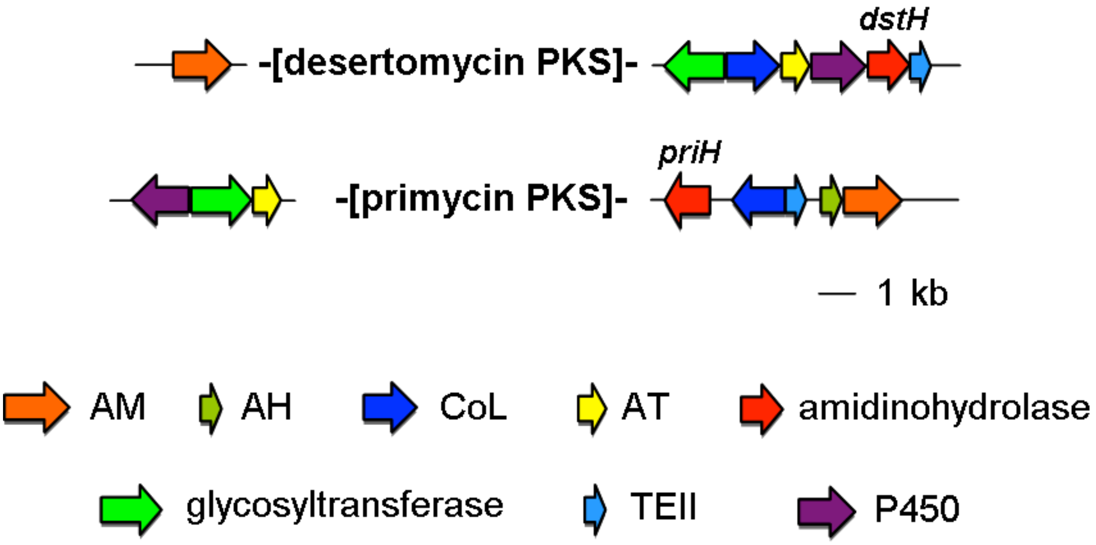

Scheme S1. Conserved genes in the desertomycin and primycin biosynthetic gene clusters. The putative amidinohydrolases encoded by genes *dstH* and *priH* are highlighted. PKS, polyketide synthase multienzymes; AM, arginine 2-mono-oxygenase; AH, 4-guanidinobutyramide hydrolase; CoL, 4-guanidinobutanoate:CoA ligase; AT, 4-guanidinobutryl-CoA:ACP acyltransferase; ACP, acylcarrier protein; TEII, discrete thioesterase.

Desertomycin

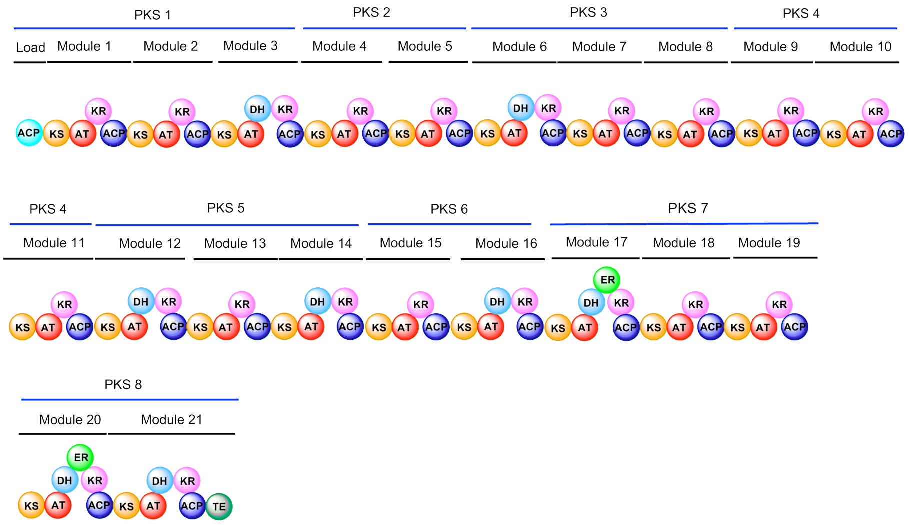

Primycin

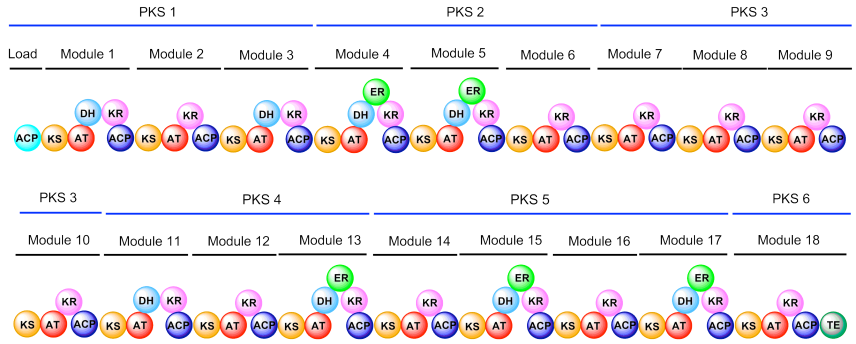

Kanchanamycin

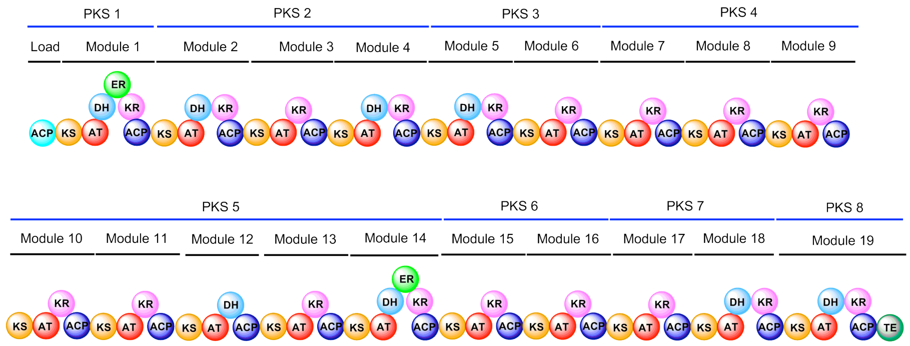

Azalomycin

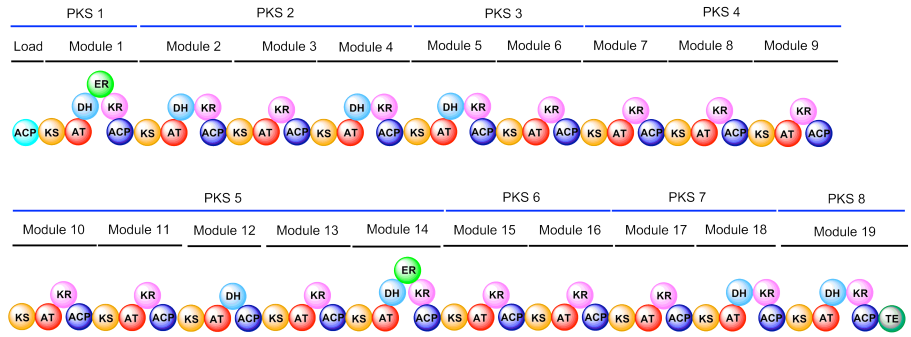

Figure S1: Polyketide synthase (PKS) domain organisations of biosynthetic gene clusters for desertomycin in Streptomyces macronensis (or in Streptomyces spectabilis NRRL B-2494, or in Streptomyces olivaceus Tü4018), primycin in **S*accharomonospora azurea DSM 43044, kanchanamycin in Streptomyces olivaceus Tü4018 and azalomycin in Streptomyces violaceusniger DSM 4137.***

Azalomycin

```
              ↓                                               ↓
KR14    HAAGVTLAASLLETELADAATVVSGKVAGAVNLDELLGDRELDAFVVFSSISGVWGGGSQGVYGSGNAFLD   A1
KR9     HAAGVEQAAELAQMGLTDAASVVSGKATGAGHLDALLGDRELDAFVVFSSIAGVWGSGGQAAYGAANAYLD   A1
KR7     HAAGVEQAAELAGMGLADAASVVSGKATGAGHLDALLGDRELDAFVVFSSIAGVWGSGGQAAYGAANAYLD   A1
KR11    HAAGANAAGPLAETTVADAAAVISGKVAGAVNLDALLGDRELDAFVVFSSIAGVWGSGGQAAYGAANAYLD   A1
KR1     HAAGVLDDGVIDTLSPKRIDAVFEPKVDAAWNLHELTRTLDLAEFVMFSSVAGVFGSPGQGNYAAANSFLD   B1
KR4     HTAGVLDDGVVEALTPERVDRVLRPKVDAVLNLHELTAGLDLSAFVLFSSLSGTLGGTGQANYAAANAFLD   B1
KR13    HAAGVLDDGVVESLTPERVDKVLRPKVDAALHLHELTRDLDLSAFILFSSVSGTFGGAGQANYAAGNAFMD   B1
KR17    HAAGVLDDGVVESLTPERVDKVLRPKVDAALHLHELTRDLDLSAFVVFSSASSNFGGGGQANYAAANAFLD   B1
KR18    HATGVLDDGLFASMTRERVDPVLRAKVDAAWNLHELTAGMDLSAFVLFSSAAGVFGSAGQSNYAAANVFLD   B1
KR2     HAAGVLDDGLLTSLTRERVEPVLRAKVDAAWNLHELTAGMDLSAFVLFSSATGVLGGAGQSNYAAANVFLD   B1
KR5     HAAGVLDDGLLTSLTRERVEPVLRAKVDAAWNLHELTAGMDLSAFVLFSSAAGTLGGPGQGSYAAANVFLD   B1
KR15    HAAGVLDDGVLDSMSVERVAGVLRPKVAGARHLHELTEGLDLSAFVLFSSLAGAIGGAGQGSYAAANAYLD   B1
KR8     HAAGVLDDGVLDGLTVEQLAGVLGAKVEGARLLHELTADLELDAFVLFSSFAGVVGGAGQGAYAAANAYLD   B1
KR10    HAAGILDDGVLDGLTVDQLAGTLAAKADGARHLHELTAELSLDAFVLFSSFAGAIGGAGQAAYAVANAYLD   B1
KR12    HAAGVLDDGLIDTLTVPRTQGVFRPKVDAVVNLHELTRDLDLSAFILFSSYAGTVGGAGQGSYAAANAFLD   B1
KR16    HTAGVLDDGVVDALTVRRAAGVLRPKVDATRNLHELTAGMDLSAFVLFSSGAATLGGPGQGSYAAGNAYLD   B1
KR3     HTAGVLDDGVVDALTVERAAGVLRPKVDAAWNLHELTAGMDLSAFVLFSSAAGTLGGPGQGSYAAGNAYLD   B1
KR6     HTAGVLDDGVLDALTVGRAAGVLRPKVDAAWNLHELTAGMDLSAFVLFSSAAGTLGGPGQGSYAAGNAYLD   B1
                                                                      *
```

Kanchanamycin

```
              ↓                                               ↓
KR14    HAAGVTLAASLLETELADAATVVSGKVAGAVNLDELLGDRELDAFVVFSSISGVWGGGSQGVYGSGNAFLD   A1
KR7     HAAGVEQAAELAGMGLADAASVISGKATGAGHLDALLGDRELDAFVVFSSIAGVWGSGGQAAYGAANAYLD   A1
KR9     HAAGVEQAAELAGMGLADAASVIAGKATGAGHLDALLGDRELDAFVVFSSIAGVWGSGGQAAYGAANAYLD   A1
KR11    HAAGANAAGPLAETTVADAAAVISGKVAGAVNLDALLGDRELDAFVVFSSIAGVWGSGGQAAYGAANAYLD   A1
KR15    HAAGVLDDGVLDSMSVERVAGVLRPKVDGARHLHELTQGLDLSAFVLFSSLAGAIGGAGQGSYAAANAYLD   B1
KR8     HAAGVLDDGVLDGLTVHQLAGVLGAKVEGARLLHELTADLELDAFVLFSSFAGVVGGAGQGAYAAANAYLD   B1
KR10    HAAGILDDGVLDGLTVGQLAGTLAAKAEGARHLHELTAELPLDAFVLFSSFAGAIGGAGQAAYAAANAYLD   B1
KR12    HAAGVLDDGLVDTLTVPRTQGVFRPKVDAVVNLHELTQDLDLSAFILFSSYAGTVGGAGQGSYAAANAFLD   B1
KR6     HTAGVLDDGVVDALTVERAAGVLRPKVDAARNLHELTAGMDLSAFVLFSSAAGTLGGPGQGSYAAGNAYLD   B1
KR3     HTAGVLDDGVVDALTVERAAGVLRPKVDAARNLHELTAGMDLSAFVLFSSGAATLGGPGQGSYAAGNSYLD   B1
KR16    HTAGVLDDGVVDALTVERAAGVLRPKVDAARNLHELTAGMDLSAFVLFSSGAATLGGPGQGSYAAGNSYLD   B1
KR18    HAAGVLDDGLFASLTRERVSAVLRAKVDAAWNLHELTADMDLSAFVLFSSAAGVLGAAGQSNYAAANVFLD   B1
KR2     HAAGVLDDGLLTSLTRERVEPVLRAKVDAAWNLHELTAGLDLSAFVLFSSAAGVLGGAGQSNYAAANVFLD   B1
KR5     HAAGVLDDGLLTSLTRERVEPVLRAKVDAAWNLHELTAGLDLSAFVLFSSAAGVLGGAGQSNYAAANVFLD   B1
KR1     HAAGVLDDGVIDTLSPKRIDAVFEPKVDAAWNLHELTRETDLAEFVMFSSVAGVFGSPGQGNYAAANSFLD   B1
KR4     HTAGVLDDGVVEALTPERVDRVLRPKVDAVLNLHELTAGLDLSAFVLFSSLSGTLGGTGQANYAAANAFLD   B1
KR13    HAAGVLDDGVVESLTPERVDRVLRPKVDAALHLHELTRDLDLSAFILFSSVSGTFGGAGQANYAAGNAFMD   B1
KR17    HAAGVLDDGVVESLTPERVDRVLRPKVDAALHLHELTRDLDLSAFVVFSSASSNFGGGGQANYAAANAFLD   B1
                                                                      *
```

Desertomycin

```
              ↓                                               ↓
```

```
KR17    HVAGVVDDGVVTALTPERLDAVLRPKVDAAVNLHELTAGL----DLSAFVLFSSAAGVLGSAGQANYAAANAFLD   B1
KR20    HVAGVVDDGVVTALTPERLDAVLRPKVDAAVNLHELTAGL----DLSAFVLFSSAAGVLGSAGQANYAAANAFLD   B1
KR12    HVAGALDDGVVTALTPERLDTVLRPKADAALHLHELSAGL----NLHAFVLFSSAAGVFGTPGQANYAAANAFLD   B1
KR3     HVAGVLDDGVVTSLTPERLDTVLRPKAEAALHLHELTAGL----DLSAFVLFSSAAGVLGSAGQANYAAANCLLD   B1
KR14    HVAGVLDDGVVTSLTPERLATVLRPKVDAARNLHELTAGL----DLSAFVLFSSASGVFGGPGQANYAAANAYLD   B1
KR16    HVAGVLDDGVVTSLTPERLARVLRPKVDAAITLHELTADL----DLSAFVLFSSASGVFGGPGQANYAAANAYLD   B1
KR6     HTAGVFDDGITASLTTEQLERVLRPKVDAAVNLHALTHDA----DLAAFVLFSSVAGVLGGAGQGNYAAGNTFLD   B1
KR21    HTAGVLDDALVASLTPERVDAVLRPKLDAALNLAELTAGH----DLAEFVLFSSAAATLGSPGQGNYAAANAFLD   B1
KR18    HAAALIELAPLATTTLGDFAEIVAAKVAGAVVLDELLSEGERAADLDAFVLFSSIAGVWGSGDHAAYAAANAHLD   A2
KR7     HAAGVGQEQPLEAMTPGDIAGVLEAKVAGAAHLDALLDTG----SLDAFVLFSSNAGVWGSASQGAYAAANAHLD   A1
KR19    HAAGVGQGQPLEETTTADIAGVLDAKVAGAQHLDALLDAG---AGLDAFVLFSSNAGVWGSGSQGAYAAANAHLD   A1
KR10    HAAGVSPALALADTTPADLAHALDAKAAGAAHLDELLDDG----ALDAFVLFSSIAAVWGSGGQAGYAAANAFLD   A1
KR13    HAAGIGQTQPLDGMGVADIAEVFGAKTAGAAHLDDLLGAD----DLDAFVLFSSNSGVWGGGGQGAYAAANAYLD   A1
KR15    HAAGVSPAHTVADMTVADIAEVFGAKTAGAAHLDDLLGAD----DLDAFVLFSSNSGVWGGGGQGAYAAANAYLD   A1
KR5     HAAGLGQDRVIGETGPEEFAAIVTAKTAGAAHLDELLGDT----PLDAFVLFSSIAGVWGSGGQAAYAAANAYLD   A1
KR2     HTAGVLDDGVLDGLTAERFATVFRPKAQAALNLHELTRDN---EHLTAFVLFSSVAGSLGIGGQGNYAAANAFLD   B1
KR1     HTAGVLDDGVVDGLTPDRLDGVLRPKSPAATALHELTRDL----DLDAFVLYSSASGALGSAGQANYAAANAHLD   B1
KR11    HAAGVLDDGVLDAMTPQRLATVFRAKAESARNLDELTADI----DLSAFVLFSSFAGVAGGAGQGSYAAANAFLD   B1
KR4     HTAGVLDDGVLGALTDDRFASVFRAKAESARHLDELTRDA----DLSAFVLFSSLTGTVGAPGQGNYAAANAYLD   B1
KR8     HTAGVLDDGVLDGLTPERLATVFRAKVESARHLDELTRDA----NLSAFVLFSSFAGLSGGTGQGSYAAANACLD   B1
KR9     HTAGVLDDGVLDGLTGERLATVFRAKVESARHLDELTRAA----DLSAFVLFSSFAGVAGGTGQGSYAAANAALD   B1
                                                                          *
```

Primycin

```
KR1     HTAAVLDDGLVTALTPQRLSAALRPKVDGAFHLHELTRDRDLTAFVLFSSVAGTVGGAGVANYAAANAFLD   B1
KR3     HTAAVLDDGLVTSFTPERVDTTLRPKADAALHLHELTRDRDLAAFVLFSSGAAVYGSKGQANYAAANSVLD   B1
KR11    HTAGALDDGLIGDLTPERVSTVLRAKADSALHLDALTRDADLSLFLLYSGAAGIFGGAGQANYAAANVLLD   B1
KR17    HTAGTLDDGLVDNLTPERVSTVLRSKVDGAVHLDELTRESDLSLFVLYSGAAGVFGGAGQSSYAAANVFLD   B1
KR4     HAAGVLDDGVVTALDRDRLARVLRPKAEAAQVLHELTRHRDLAQFVLFSSGAGVFGSPGQGNYAAANAFLD   B1
KR13    HTAGVLDDGVVESLTAQRVRKVMDPKASAAWNLHELTRDRDVAEFVLFSSASGIFGNAGQANYSAANTFLD   B1
KR5     HTAGVLDDGVVESMTAQRIETVMAPKALAAWNLHELTRDRDVAEFVLFSSASGVFGNAGQANYSAANTFLD   B1
KR15    HTAGVLDDGVVESMTAQRIETVMAPKALAAWNLHELTRDHDVAEFVLFSSASGVFGNPGQANYSAANAFLD   B1
KR2     HVAAVLDDSLIDSLTVEQIHRVAGVKVGGTLNLHELTADMPLSAFVVFSSFAGTTGGPGQGNYAPGNAFLD   B2
KR7     HTAGVLDDGVLEHMSAEQFTGVLGAKADAALWLHELTRDLDLTAFVLFSAFAGTVGSAGQGNYAAANAVLD   B1
KR9     HAAGVLDDGVIEGLTPDRVRGVLRAKVDGATLLHELTGDL--DAFVVFSAFAGAIGSAGQASYAAANAHLD   B1
KR18    HTAGIAPSIPLEETTPEVLAEVYAGKVTGAELLDELLADTALDAFVLFSSCAGVWGGIGQAAYAAANAHLD   A1
KR8     HAAGAAQVTPLTDIGPAEFAEVVAAKVLGARHLHELTEDL--SAFVVFSSIAATWGSGGQSAYAVANAYLD   A1
KR6     HAAGVSQSTPLADTTPEEFAAVVAGKVAGAMHLHELTADL--DAFVVFSSIAGVWGSAGQVAYSAANAALD   A1
KR10    HAAGVAQSTPLVECTAEEFENVMSGKVAGARNLHEATKELPLLAFIVFSSIAATWGSGGQCGYAAGNAFLD   A1
KR12    HAAGIAQSTPLVDCSVEEFAEVVAGKVAGAVNLHELTEDL--DAFIVFSSIAATWGSGGQCGYAAGNAFLD   A1
KR14    HAAGVAQSTPLVECSVEEFAEVVAGKVAGAVNLHELTGEL--DAFIVFSSIAATWGSGGQCGYAAGNAFLD   A1
KR16    HAAGMAQSTALVDCSVEEFAEVVAGKVAGAVNLHELTEDL--DAFIVFSSIAATWGSGGQCGYAAGNAFLD   A1
                                                                      *
```

Figure S2: Sequence alignment of the PKS KR domains. The active site residue Y is marked with an asterisk. The arrows indicate the residue predictive of B and A-type alcohol stereochemistry, respectively. The predicted configurations of the α- and β-stereocenters generated by each KR, according to the model of Keatinge-Clay,[6] are indicated to the right of the alignment. A1: *2R*, *3S*; A2: *2S*, *3S*; B1: *2R*, *3R*; B2: *2S*, *3R*.

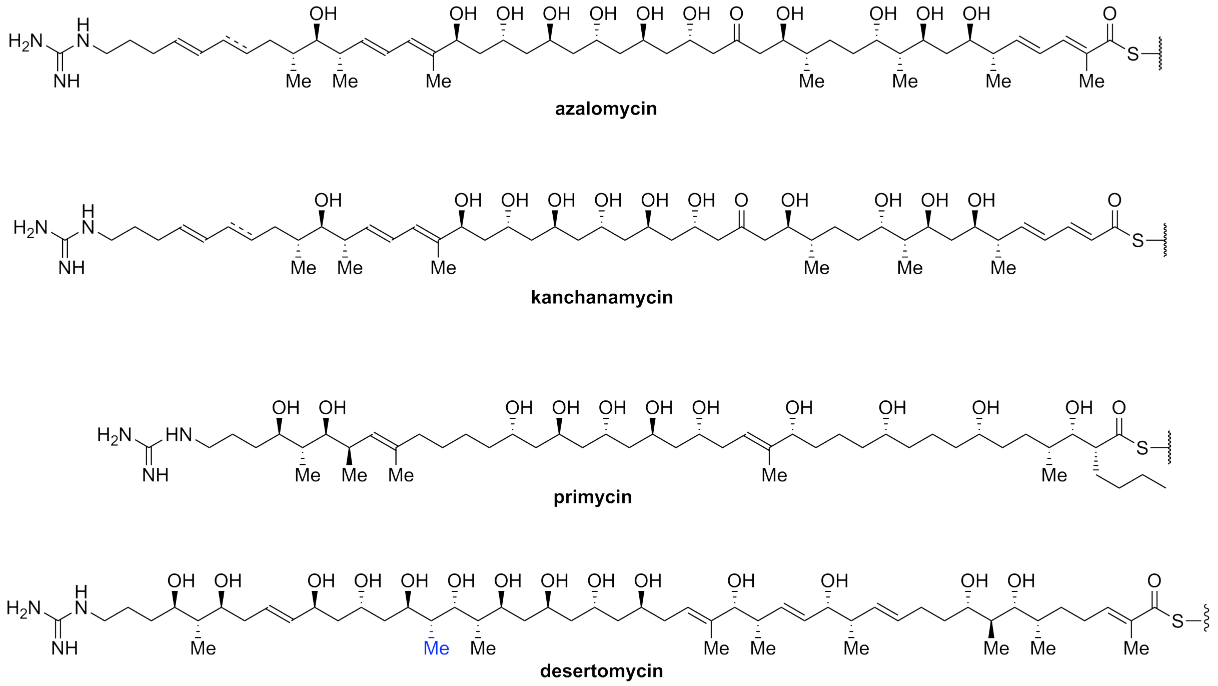

Figure S3: Predicted linear structures for azalomycin, kanchanamycin, primycin and desertomycin based on the bioinformatic analysis of ketoreductase (KR) domain. The predicted configuration for desertomycin at C32-methyl (highlighted in blue) is opposite from the configuration established by Kishi et al.,[7] all the other 20 stereocenters have the same configurations as those established by Kishi and colleagues.

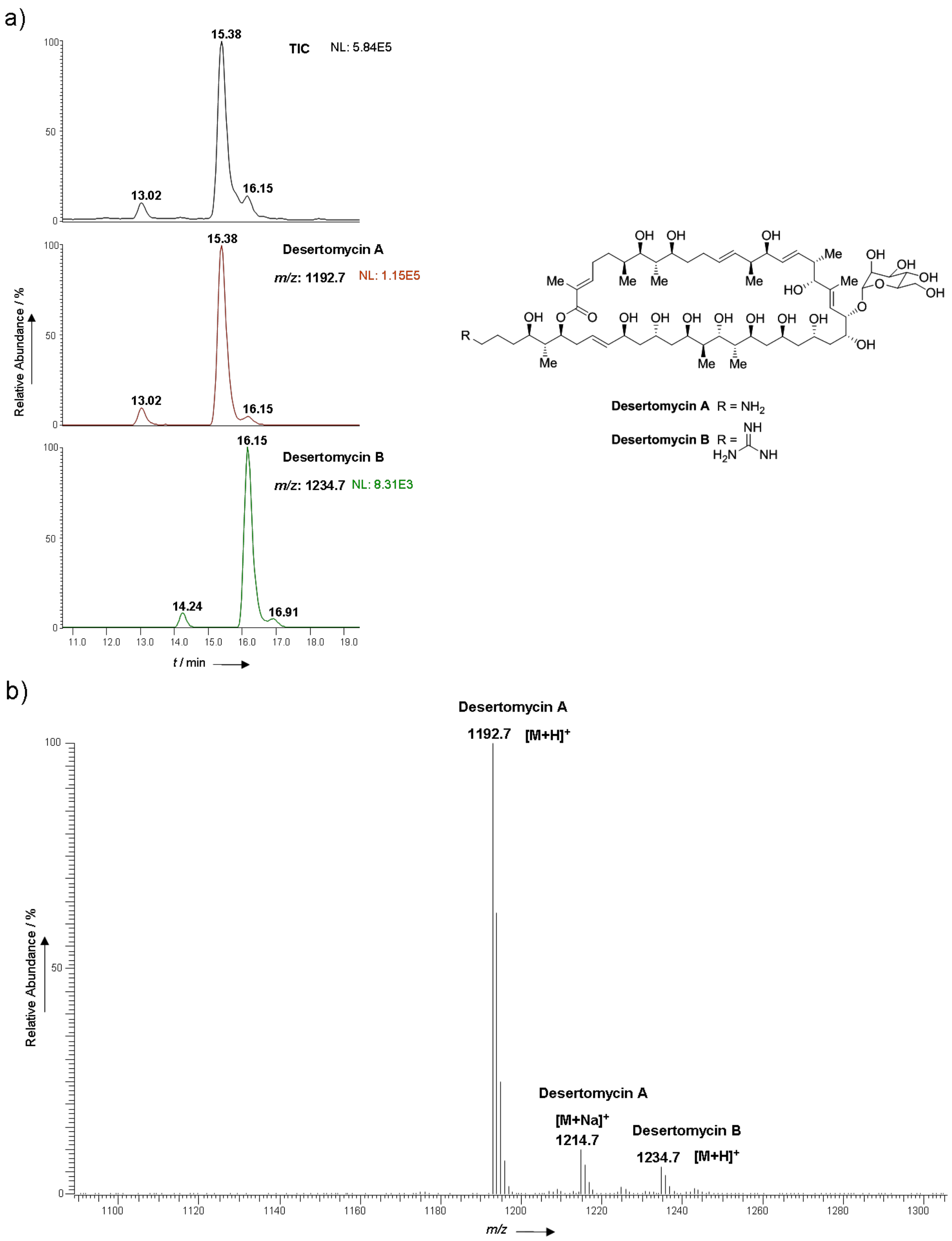
a)
TIC
NL: 5.84E5
15.38
13.02
16.15
Desertomycin A
m/z: 1192.7
NL: 1.15E5
Desertomycin B
m/z: 1234.7
NL: 8.31E3
14.24
16.91
Relative Abundance / %
t / min
Desertomycin A R = NH2
Desertomycin B R =
b)
Desertomycin A
1192.7 [M+H]+
Desertomycin A
[M+Na]+
1214.7
Desertomycin B
1234.7 [M+H]+
Relative Abundance / %
m/z

c)

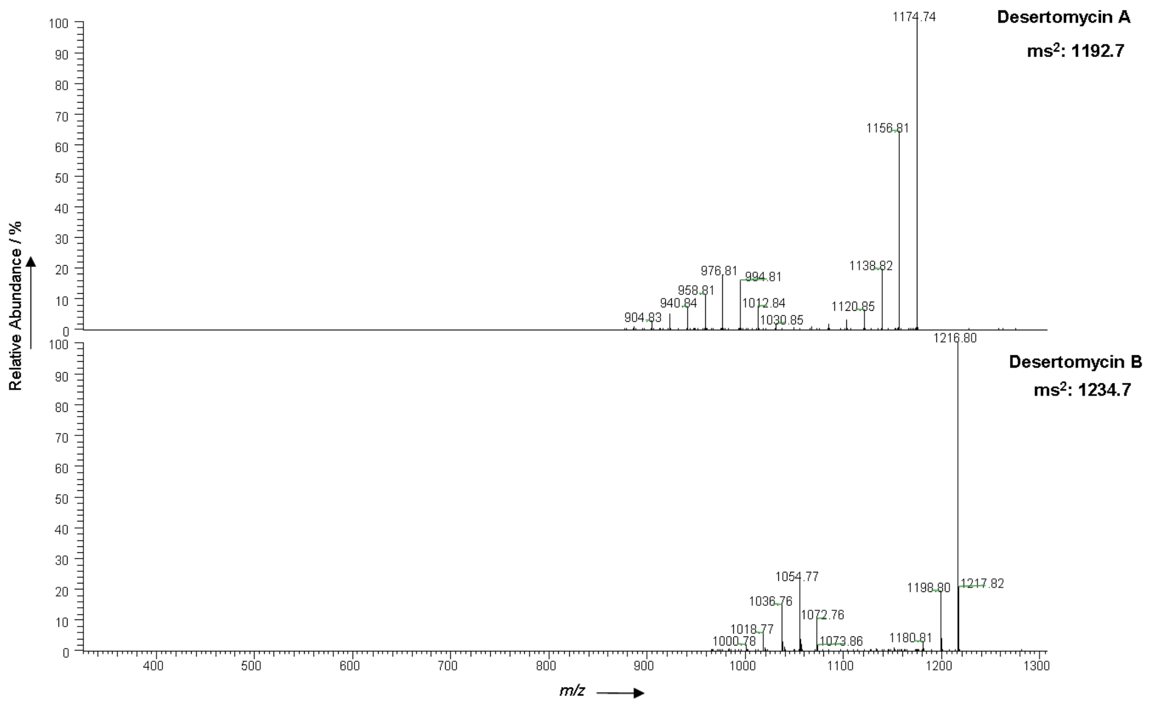

Figure S4. HPLC-MS analysis of desertomycin production in S. macronensis. a) LC-MS chromatogram of desertomycin A and desertomycin B. b) ESI-MS spectrum of desertomycin A ([M+H]$^+$: 1192.7; [M+Na]$^+$: 1214.7) and desertomycin B ([M+H]$^+$: 1234.7). c) ESI-MS/MS spectra of desertomycin A ([M+H]$^+$: 1192.7) and desertomycin B ([M+H]$^+$: 1234.7).

a)

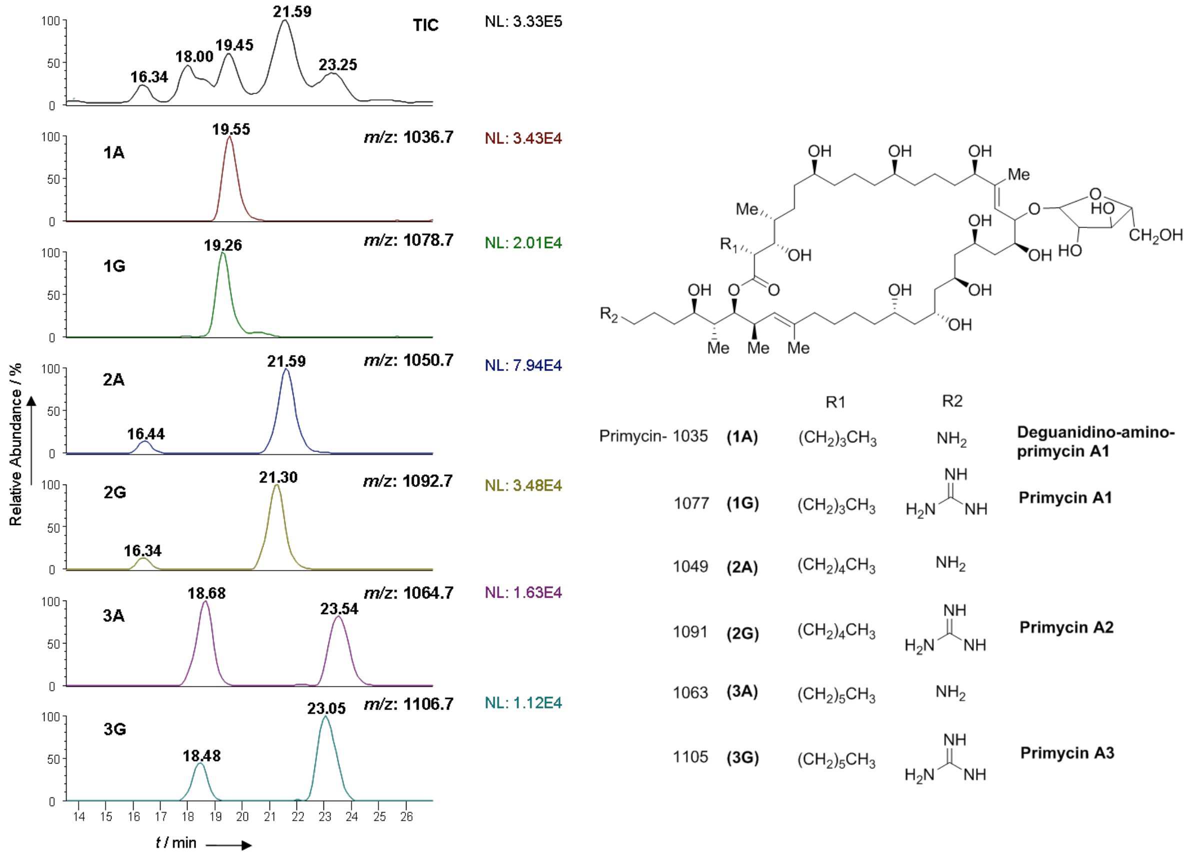

TIC
NL: 3.33E5
21.59
19.45
18.00
16.34
23.25
1A
19.55
m/z: 1036.7
NL: 3.43E4
1G
19.26
m/z: 1078.7
NL: 2.01E4
2A
21.59
16.44
m/z: 1050.7
NL: 7.94E4
2G
21.30
16.34
m/z: 1092.7
NL: 3.48E4
3A
18.68
23.54
m/z: 1064.7
NL: 1.63E4
3G
23.05
18.48
m/z: 1106.7
NL: 1.12E4
Relative Abundance / %
t / min
R1
R2
Primycin- 1035 (1A) (CH2)3CH3 NH2 Deguanidino-amino-primycin A1
1077 (1G) (CH2)3CH3 Primycin A1
1049 (2A) (CH2)4CH3 NH2
1091 (2G) (CH2)4CH3 Primycin A2
1063 (3A) (CH2)5CH3 NH2
1105 (3G) (CH2)5CH3 Primycin A3

b)

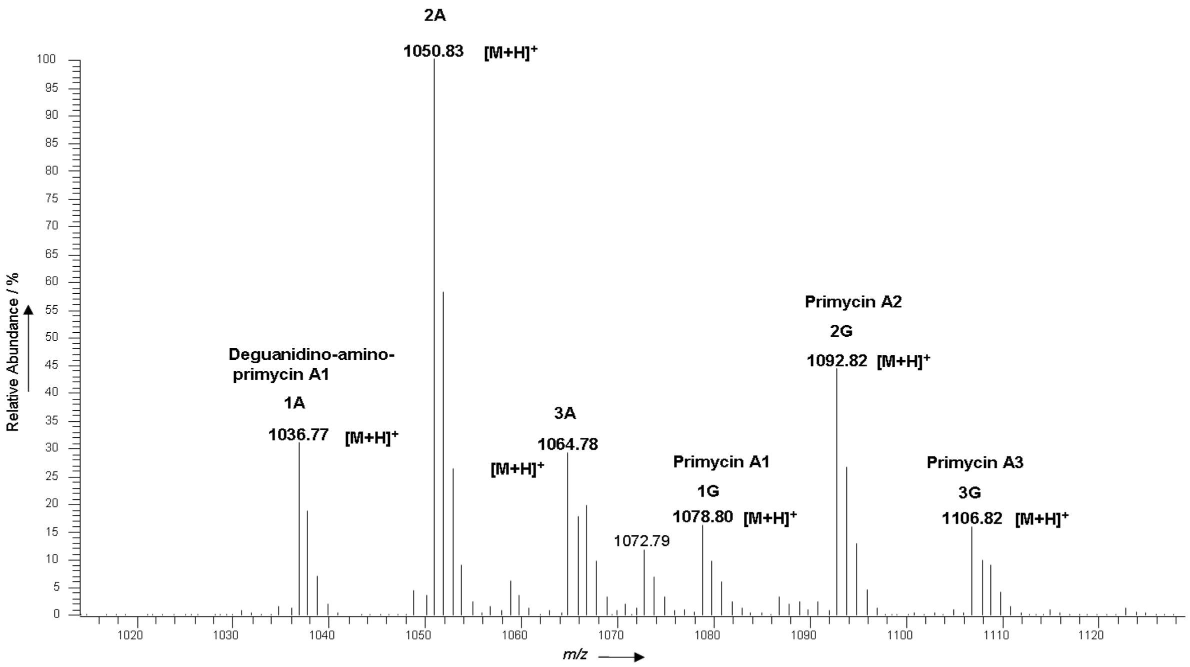

2A
1050.83 [M+H]+
Deguanidino-amino-primycin A1
1A
1036.77 [M+H]+
3A
1064.78
[M+H]+
1072.79
Primycin A1
1G
1078.80 [M+H]+
Primycin A2
2G
1092.82 [M+H]+
Primycin A3
3G
1106.82 [M+H]+
Relative Abundance / %
m/z

c)

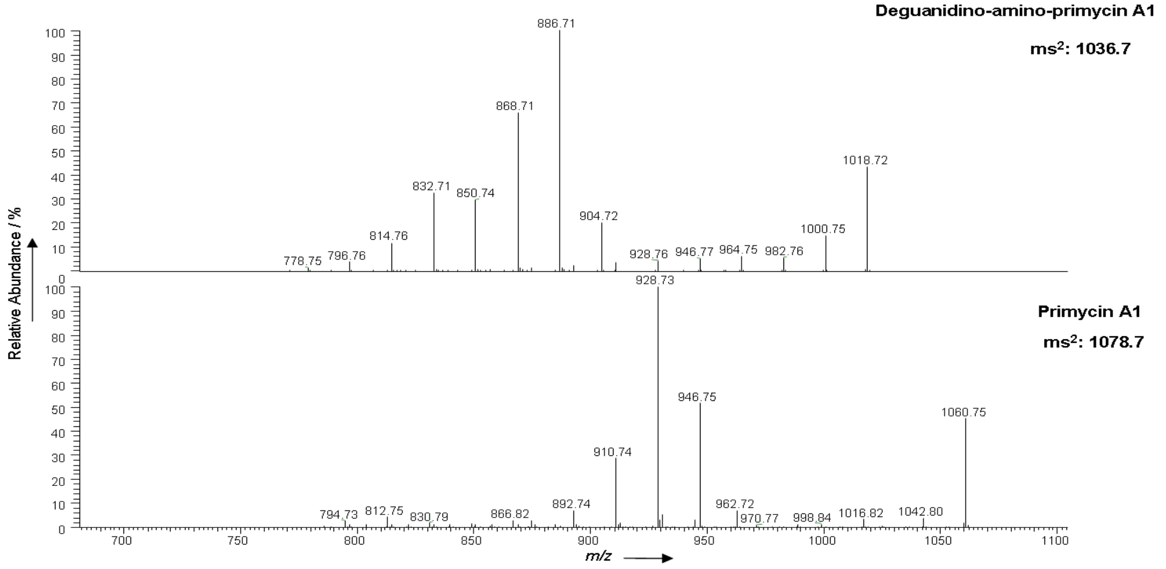

Figure S5. HPLC-MS analysis of primycins in Saccharomonospora azurea. a) LC-MS chromatogram of various primycins. b) ESI-MS spectrum of primycins. c) ESI-MS/MS spectra of deguanidino-amino-primycin A1 ($[M+H]^+$: 1036.7) and primycin A1 ($[M+H]^+$: 1078.7). The isomers for **2A**, **2G**, **3A** and **3G** likely represent structural isomers of side-chain R_1.

a)

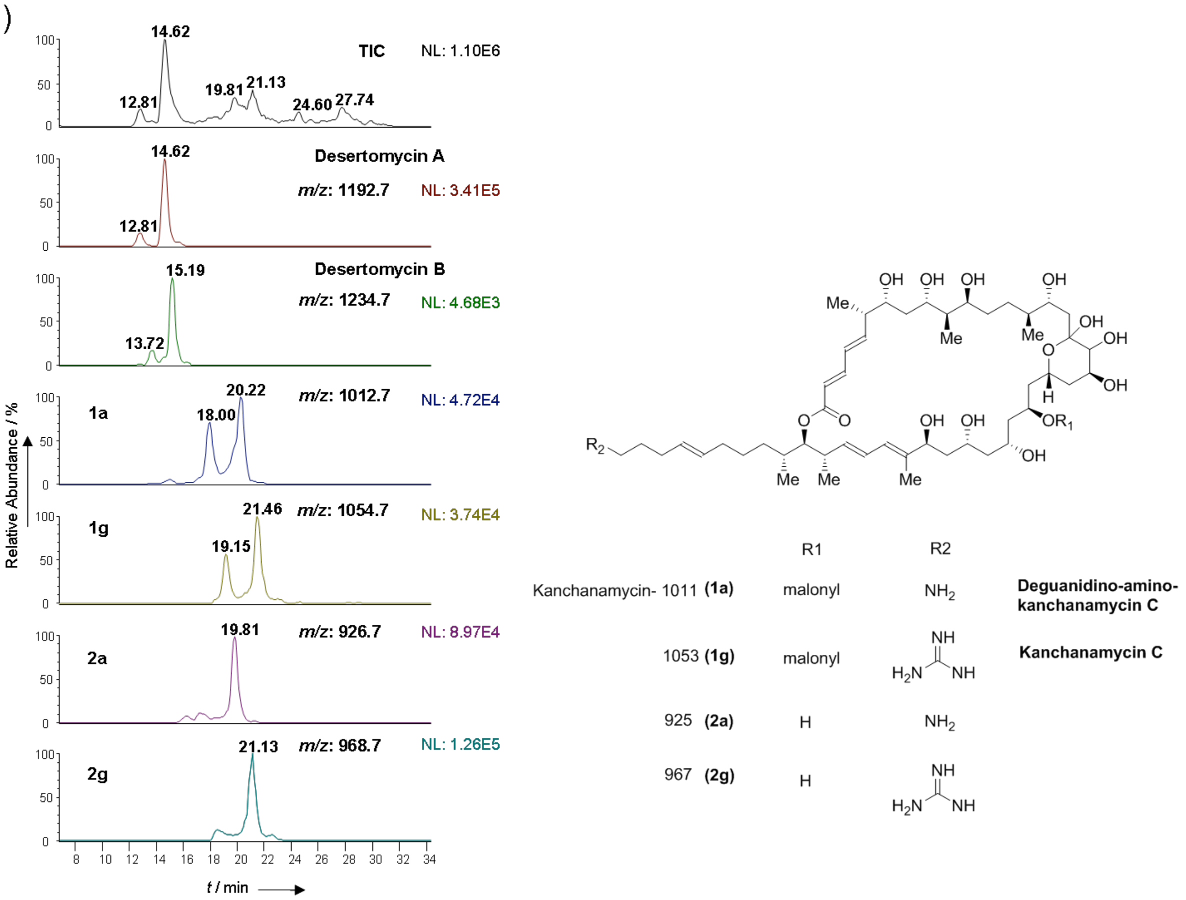

b)

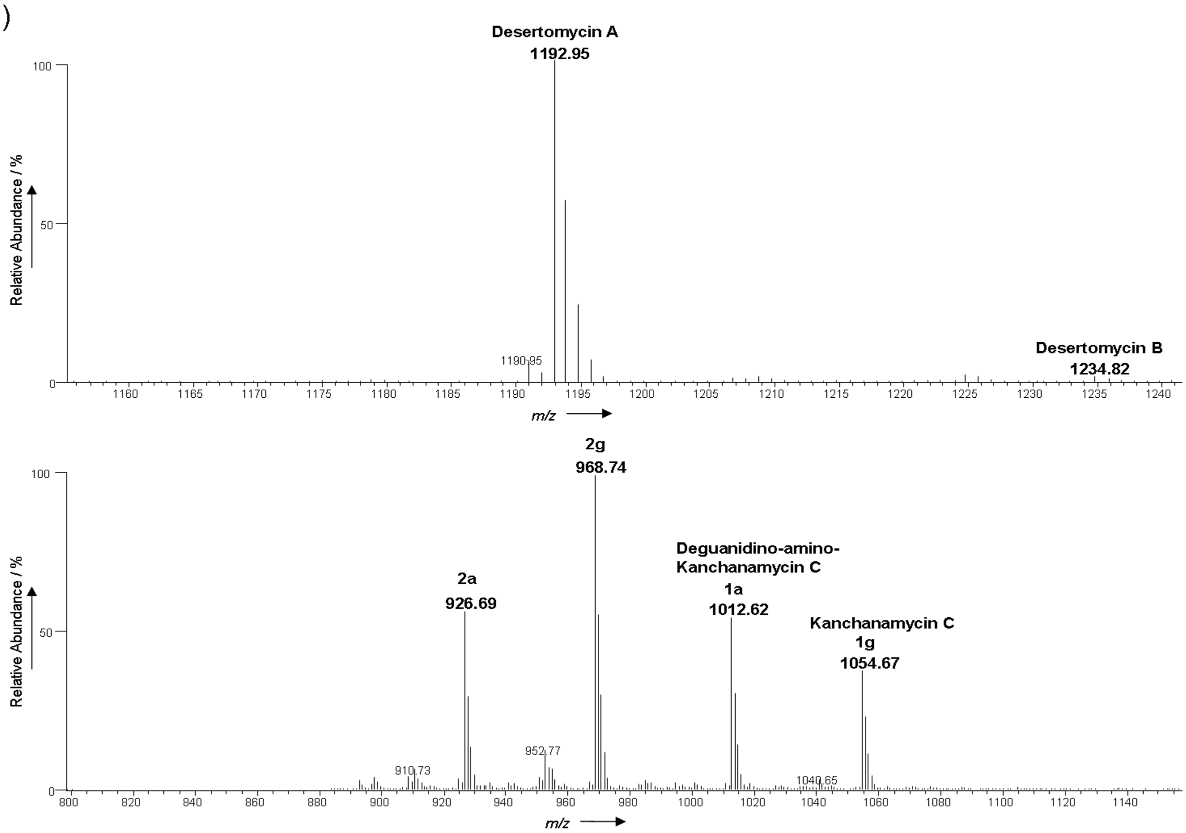
Desertomycin A
1192.95
Desertomycin B
1234.82
Relative Abundance / %
m/z
2g
968.74
2a
926.69
Deguanidino-amino-
Kanchanamycin C
1a
1012.62
Kanchanamycin C
1g
1054.67
910.73
952.77
1040.65
1190.95

c)

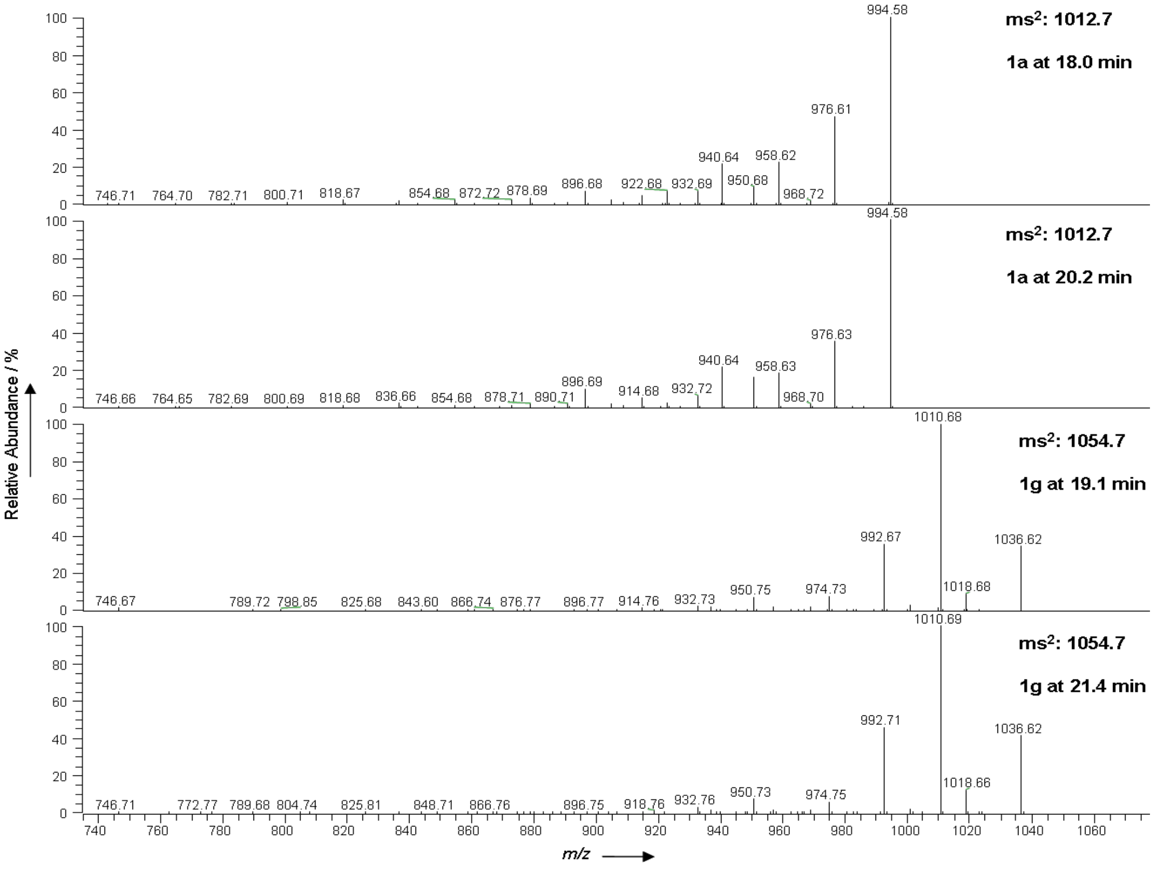
ms²: 1012.7
1a at 18.0 min
ms²: 1012.7
1a at 20.2 min
ms²: 1054.7
1g at 19.1 min
ms²: 1054.7
1g at 21.4 min
994.58
976.61
976.63
1010.68
1010.69
992.67
992.71
1036.62
1018.68
1018.66
Relative Abundance / %
m/z

Figure S6. HPLC-MS analysis of desertomycins and kanchanamycins in Streptomyces olivaceus Tü4018. a) LC-MS chromatogram of desertomycin A, desertomycin B and kanchanamycins. b) ESI-MS spectra of desertomycin A, desertomycin B and kanchanamycins. c) ESI-MS/MS spectra of deguanidino-amino-kanchanamycin C ($[M+H]^+$: 1012.7) and kanchanamycin C ($[M+H]^+$: 1054.7). The two peaks for deguanidino-amino-kanchanamycin C at 18.0 min and 20.2 min are probably isomers, the same is true for kanchanamycin C at 19.1 min and 21.4 min. The two isomers, by analogy with azalomycin, are likely due to a different site of attachment of the malonyl group, either at C23-OH or at C25-OH.[8]

a)

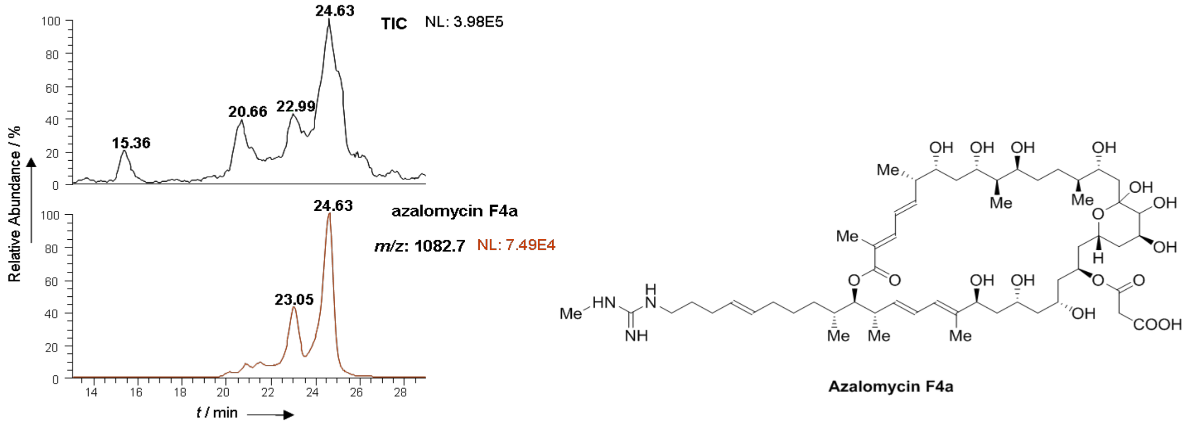

b)

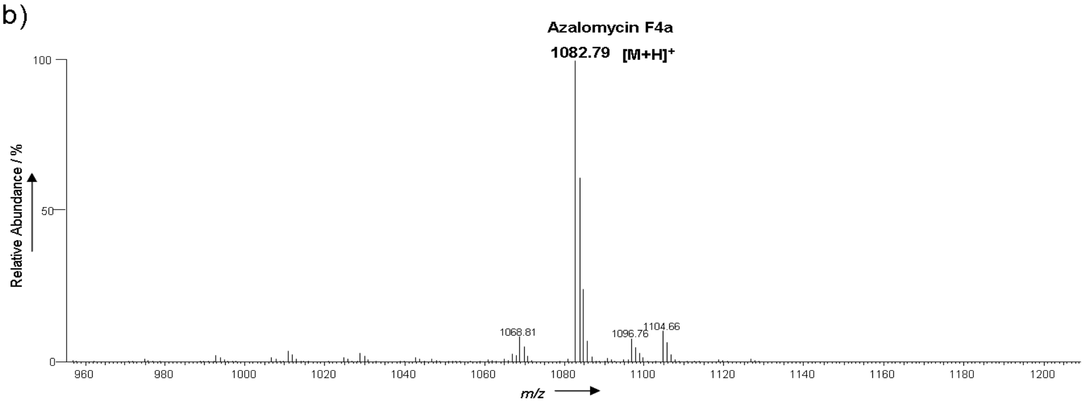

c)

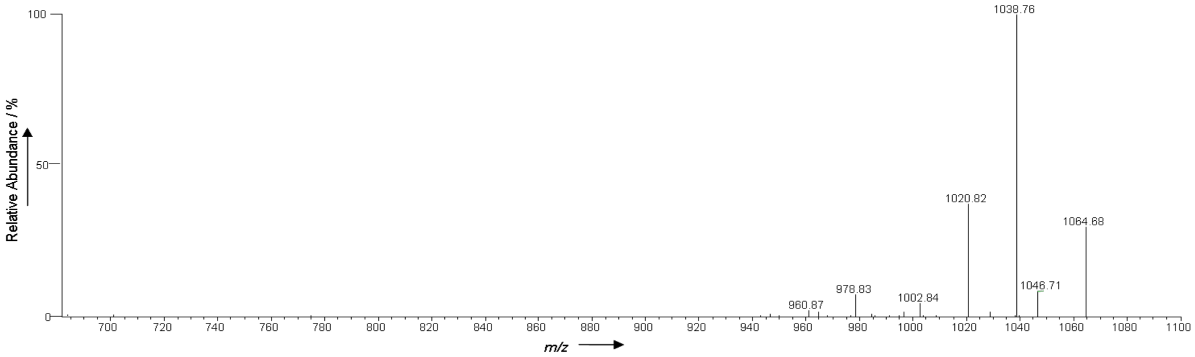

Figure S7. HPLC-MS analysis of azalomycin F4a in Streptomyces violaceusniger DSM4137. a) LC-MS chromatogram of azalomycin F4a. b) ESI-MS spectrum of azalomycin F4a. c) ESI-MS/MS spectrum of azalomycin F4a ($[M+H]^+$: 1082.7). The two peaks for azalomycin F4a at 23.0 min and 24.6 min are isomers, which are due to different attachment site of malonyl group, one is at C23-OH and the other is at C25-OH.[8]

A)

```
                     10        20        30        40        50        60
                      |         |         |         |         |         |
AMH_A828     --MSETPESEAWRREVDRSTFPRREPGPIDLRRYYVQPSYSGVPTFMGVPLALTQEDLRA
AMH_A821     VKSEHTDDSASWPYPIKQAS---RDPGPLNVHRNANQPAYVGIPTFMSLPICLTPEDLRA
GbuA_PA      -----------------------NLHQPLGGNE---MPRFGGIATMMRLPHVQSPAELDA
GpuA_PA      -------------------------PQPLDAAE---IPRFAGIPTFMRLPAFTDPA---A
PAH_SC       ------------------------------------SPRYAQIPTFMRLPHDPQPR---G
Agm_BT       ------------------------------------------------------------
Agm_DR       ---------------------------------------YGGIPTFARAPLVQPDGDWQA
Agm_CD       ---------------------------------------------------------LNY
Agm_TV       ------------------------------------------------------------
ARG_BC       ------------------------------------------------------------
ARG_TT       ------------------------------------------------------------

                     70        80        90       100       110       120
                      |         |         |         |         |         |
AMH_A828     GEVDVAVVGCPVDVSSGHR-GAAYGPRAIRADERYLYATPEGFVHSATRVNPFNILKVVD
AMH_A821     GDVDVAVLGAPVDTSTGHR-GAAFGPRALRADERYLFNNTSDLVNASTRIKPFDELTVVD
GbuA_PA      --LDAAFVGVPLDIGTSLRSGTRFGPREIRAESVMIRP-----YNMATGAAPFDSLNVAD
GpuA_PA      --LQVGLIGVPWDGGTTNRAGARHGPREVRNLSSLMRK-----VHHVSRIAPYDLVRVGD
PAH_SC       --YDVVVIGAPYDGGTSYRPGARFGPQAIRSESGLIHG-----VGIDRGPGTFDLINCVD
Agm_BT       ----------PLDLATTFRSGARLGPSAVRAASVQLAE----LNPYPWGFDPFDDLAVID
Agm_DR       ---DVAALGVPFDIALGFRPGARFAPRALREASLRSVP----PFTGLDGKTRLQGVTFAD
Agm_CD       EESNLIVFGVGFDGTTSNRPGARFASSSXRKEFYGLET------YSPFLDLDLEDYNICD
Agm_TV       ------VFGIPFDNTSSYRRGSKYAPDSIRGAYVNLES------YEYSYGIDLLASGXAD
ARG_BC       ----ISIIGVPMDLGQT-RRGVDMGPSAMRYAGVIERLERLHYDIEDLGDIPIG--KAER
ARG_TT       ----VAVVGVPMDLGAN-RRGVDMGPSALRYARLLEQLEDLGYTVEDLGDVPVSLARASR

                    130       140       150       160       170       180
                      |         |         |         |         |         |
AMH_A828     YGDAAVDPFDITRSMEPIRGLVREIAEV----GARPVVLGGDHSLLWPSVGALSEVHGRG
AMH_A821     YGDAAVDLWSIENTERTIGQVVSEVLDV----GAVPLVMGGDHSVMVPNVRALVEKYGAD
GbuA_PA      IGDVAINTFNLLEAVRIIEQEYDRILGH----GILPLTLGGDHTITLPILRAIXKXHG--
GpuA_PA      LGDAPVNPIDLLDSLRRIEGFYRQVHAA----GTLPLSVGGDHLVTLPIFRALGRERP--
PAH_SC       AGDINLTPFDMNIAIDTAQSHLSGLLKA----NAAFLMIGGDHSLTVAALRAVAEQHG--
Agm_BT       YGDCWFDAHHPLSIKPAIVEHARTILQS----DARMLTLGGDHYITYPLLIAHAQKYGK-
Agm_DR       AGDVILPSLEPQLAHDRITEAARQVRGR----CRVPVFLGGDHSVSYPLLRAFADVPD--
Agm_CD       YGDLEISVGSTEQVLKEIYQETYKIVRD----SKVPFXIGGEHLVTLPAFKAVHEKYN--
Agm_TV       LGDXEESE-DVEYVIDTVESVVSAVXSD----GKIPIXLGGEHSITVGAVRALPK-----
ARG_BC       LHEQGDSRLRNLKAVAEANEKLAAAVDQVVQRGRFPLVLGGDHSIAIGTLAGVAKHYE--
ARG_TT       RRGRGLAYLEEIRAAALVLKERLAALPE----GVFPIVLGGDHSLSMGSVAGAARG-R--
                                                          *◄

                    190       200       210       220       230       240
                      |         |         |         |         |         |
AMH_A828     SIAVIHFDAHPDCHEELFGHR-ATHTTPIRRLIDEE------------MVPGPNVIQVGI
AMH_A821     KLAVVHFDAHPDCHEEIYGHT-KTHATTIWRLVNEL------------GVPGHNIVQAGI
GbuA_PA      XVGLVHVDAHADVNDHMFGEX-IAHGTTFRRAVEED------------LLDCDRVVQIGL
GpuA_PA      -LGMVHFDAHSDTNDRYFGDNPYTHGTPFRRAIEEG------------LLDPLRTVQIGI
PAH_SC       PLAVVHLDAHSDTNPAFYGGR-YHHGTPFRHGIDEK------------LIDPAAMVQIGI
Agm_BT       PLSLIHFDAHCDTWADDAPDS-LNHGTMFYKAVKDG------------LIDPKASVQVGI
Agm_DR       -LHVVQLDAHLDFTDTR-NDTKWSNSSPFRRAC-EA------------LPNLVHITTVGL
Agm_CD       DIYVIHFDAHTDLREEYNNSK-NSHATVIKRIWD--------------IVGDNKIFQFGI
Agm_TV       DVDLVIVDAHSDFRSSYXGNK-YNHACVTRRALD--------------LLGEGRITSIGI
ARG_BC       RLGVIWYDAHGDVNTAETSPSGNIHGMPLAASLGFGHPALTQIGGYSPKIKPEHVVLIGV
ARG_TT       RVGVVWVDAHADFNTPETSPSGNVHGMPLAVLSGLGHPRLTEV---FRAVDPKDVVLVGV
                   * **            ▲
```

```
                 250       260       270       280       290       300
                           |         |         |         |         |         |
AMH_A828      RTISGPDDQLFNWMRRAGMRSHFMAEIERIGFAAVIDKVIEEARAVADHVYLSLDIDVLD
AMH_A821      RTPGSPDNQLFHWMRKAGIHTHFMAEIERLGLPAVVDKVIAEASDGAEVVYVSLDIDVVD
GbuA_PA       RAQGYTAED-FNWSRXQGFRVVQAEECWHXSLEPLMAEVREXV--GGGPVYLSFDIDGID
GpuA_PA       RGSVYSPDD-DAFARECGIRVIHMEEFVELGVEATLAEARRVV--GAGPTYVSFDVDVLD
PAH_SC        RGHNPKPDS-LDYARGHGVRVVTADEFGELGVGGTADLIREKV--GQRPVYVSVDIDVVD
Agm_BT        RTWNDD---------YLGINVLDAAWVHEHGARATLERIESIV--GGRPAYLTFDIDCLD
Agm_DR        RGLRFDPEA-VAAARARGHTIIPMDDV-TADLAGVLAQLPR-----GQNVYFSVDVDGFD
Agm_CD        RSGTKEE---FKFATEEKHTYXEI-----GGIDTFENIVNXL---NGKNIYLTIDLDVLD
Agm_TV        RSVSREE---FEDPDFRKVSFISSFDVKKNGIDKYIEEVDR----KSRRVYISVDXDGID
ARG_BC        RSLDEGEK---KFIREKGIKIYTMHEVDRLGMTRVMEETIAYLKERTDGVHLSLDLDGLD
ARG_TT        RSLDPGEK---RLLKEAGVRVYTMHEVDRLGVARIAEEVLKHLQGLP--LHVSLDADVLD
                                                                      * *
                        310       320       330       340       350       360
                          |         |         |         |         |         |
AMH_A828      PAFAPGTGTPEPAGLTTRELFTALRRIAH-ETNLVGMDVVEVAPHLDAGYSTAMNARRAV
AMH_A821      PAYAPGTGTPEPGGLSGREILTAFRRLCH-ELPVVGMDVVEVAPHLDPGYHTALLARRVI
GbuA_PA       PAWAPGTGTPEIGGLTTIQAMEIIRGC-Q-GLDLIGCDLVEVSPPYDTTGNTSLLGANLL
GpuA_PA       PAFAPGTGTPEIGGMTSLQAQQLVRGL-R-GLDLVGADVVEVSPPFDVGGATALVGATMM
PAH_SC        PAFAPGTGTPAPGGLLSREVLALLRCV-G-DLKPVGFDVMEVSPLYDHGGITSILATEIG
Agm_BT        PAFAPGTGTPVAGGLSSAQALAIVRGL-G-GVNLIGADVVEVAPAYDQSEITAIAAAHVA
Agm_DR        PAVIPGTSSPEPDGLTYAQGMKILAAAAA-NNTVVGLDLVELAPNLDPTGRSELLMARLV
Agm_CD        ASVFPGTGTPEPGGVNYREFQEIFKIIKNSNINIVGCDIVELSPDYDTTGVSTVIACKIL
Agm_TV        PAYAPAVGTPEPFGL---ADTDVRRLIERLSYKAVGFDIVEFSPLYDNGNTSXLAAK---
ARG_BC        PSDAPGVGTPVIGGLTYRESHLAMEMLAE-AQIITSAEFVEVNPILDERNKTASVA----
ARG_TT        PTLAPGVGTPVPGGLTYREAHLLMEILAE-SGRVQSLDLVEVNPILDERNRTAEMLVGLA
                        ▲                                ▲
                        370       380       390
                          |         |         |
AMH_A828      FEALTGLALNRIKISSKNYANPIVAGEVRFPLK----
AMH_A821      LESISGLAMRKAGISTRDYRHPVVSGEIPFAMPARRS
GbuA_PA       YEMLCVL------------------------------
GpuA_PA       FELLCLLAESAA-------------------------
PAH_SC        AE-----------------------------------
Agm_BT        CDLLCLWRQRKAG------------------------
Agm_DR        METLC--------------------------------
Agm_CD        RE-----------------------------------
Agm_TV        -------------------------------------
ARG_BC        -------------------------------------
ARG_TT        LSLLG--------------------------------
```

B)

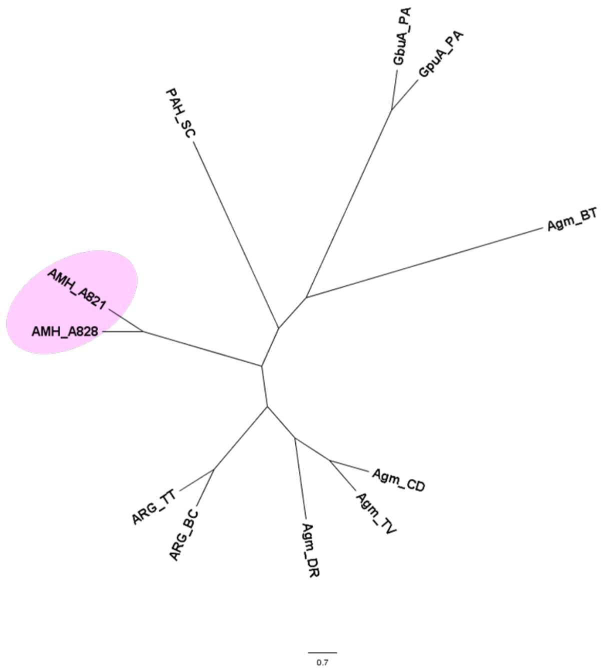

Figure S8. **A) Sequence alignment of ureohydrolases.** The sequences of eleven ureohydrolases [AMH_A828: amidinohydrolase from *Streptomyces olivaceus*Tü4018 (A828); AMH_A828: amidinohydrolase from *Saccharomonospora caesia* (A821); GbuA_PA: guanidinobutyrase from *Pseudomonas aeruginosa*; GpuA_PA: guanidinopropionase from *Pseudomonas aeruginosa*; PAH_SC: proclavaminic acid amidino hydrolase (PAH) from *Streptomyces clavuligerus*; Agm_BT: agmatinase from *Burkholderia thailandensis*; Agm_DR: agmatinase from *Deinococcus radiodurans*; Agm_CD: agmatinase from *Clostridium. difficile*; Agm_TV: agmatinase from *Thermoplasma. volcanium*; ARG_BC: Arginase from *Bacillus. caldovelox*; ARG_TT: Arginase from *Thermus. Thermophilus*] are aligned using MultAlin. Three well-conserved sequences (xGGDH, DAHxD, and SxDxDxxDPxxxP) in most of the ureohydrolases are indicated by black boxes. The metal binding sites are indicated with asterisks, guanidino ligands with black triangles. **B) Cladogram of amidinohydrolases AMH_A828, AMH_A821, and homologues.** Analyses were performed using FigTree v1.4.2.0.

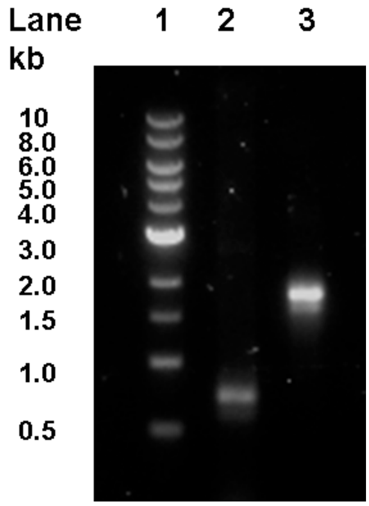

Figure S9. In-frame deletion of amidinohydrolase gene dstH in Streptomyces macronensis. Lane 1: marker; Lane 2 and 3: PCR product from ΔdstH (684 bp) and WT (1,770 bp), respectively.

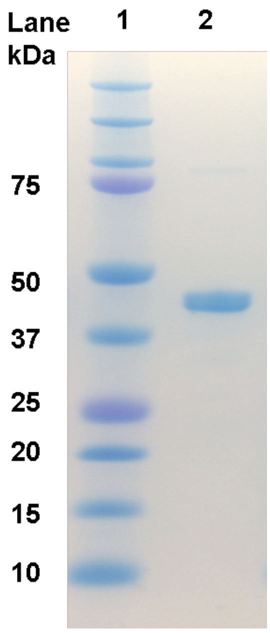

Figure S10. 4 - 12% Bis-Tris SDS-PAGE analysis of DstH. Lane 1, protein standards; Lane 2, DstH (43 kDa).

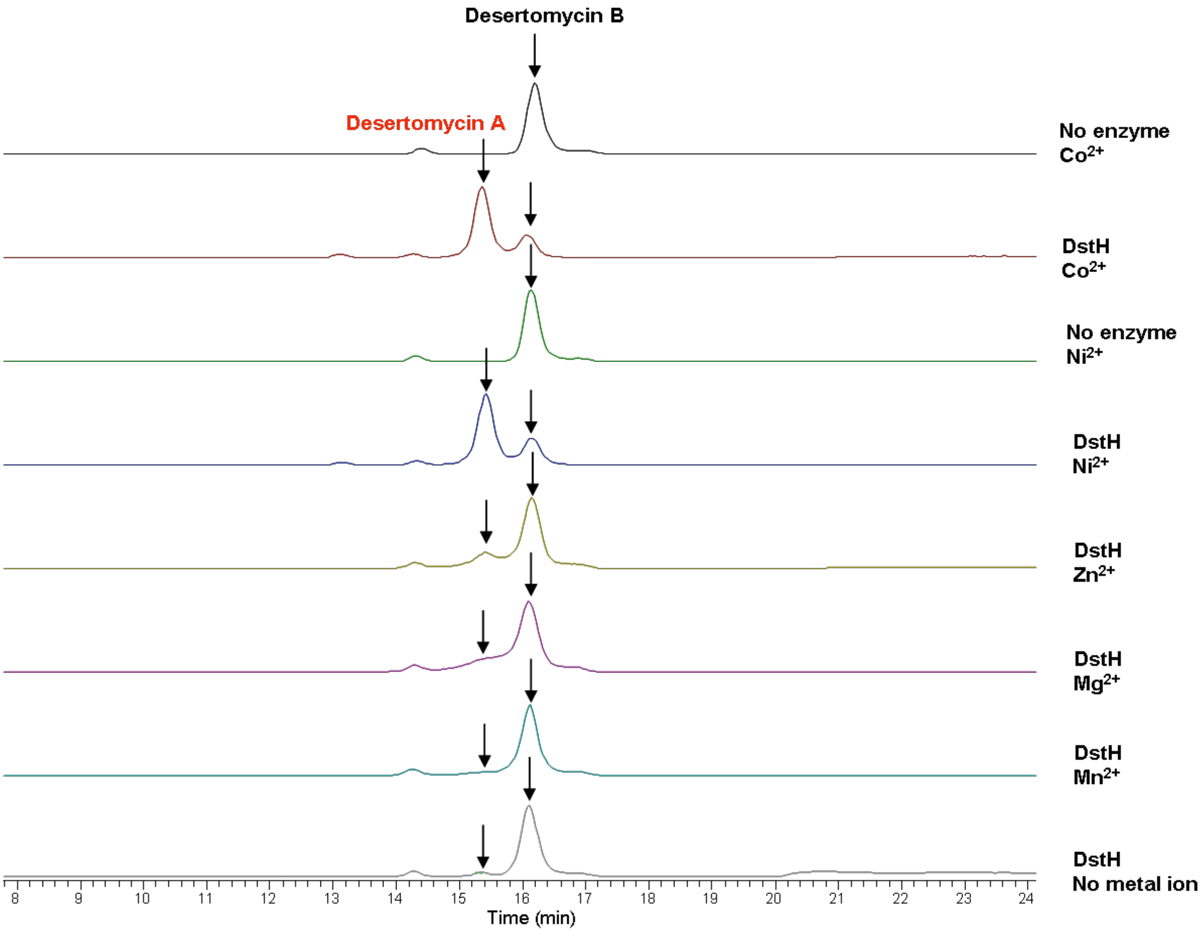

Figure S11. HPLC-ESI-MS total ion current traces of in vitro amidinohydrolysis of desertomycin B catalysed by DstH in the presence of various divalent ions. Desertomycin B is efficiently converted to its amino form 1a in the presence of either Co^{2+} or Ni^{2+}.

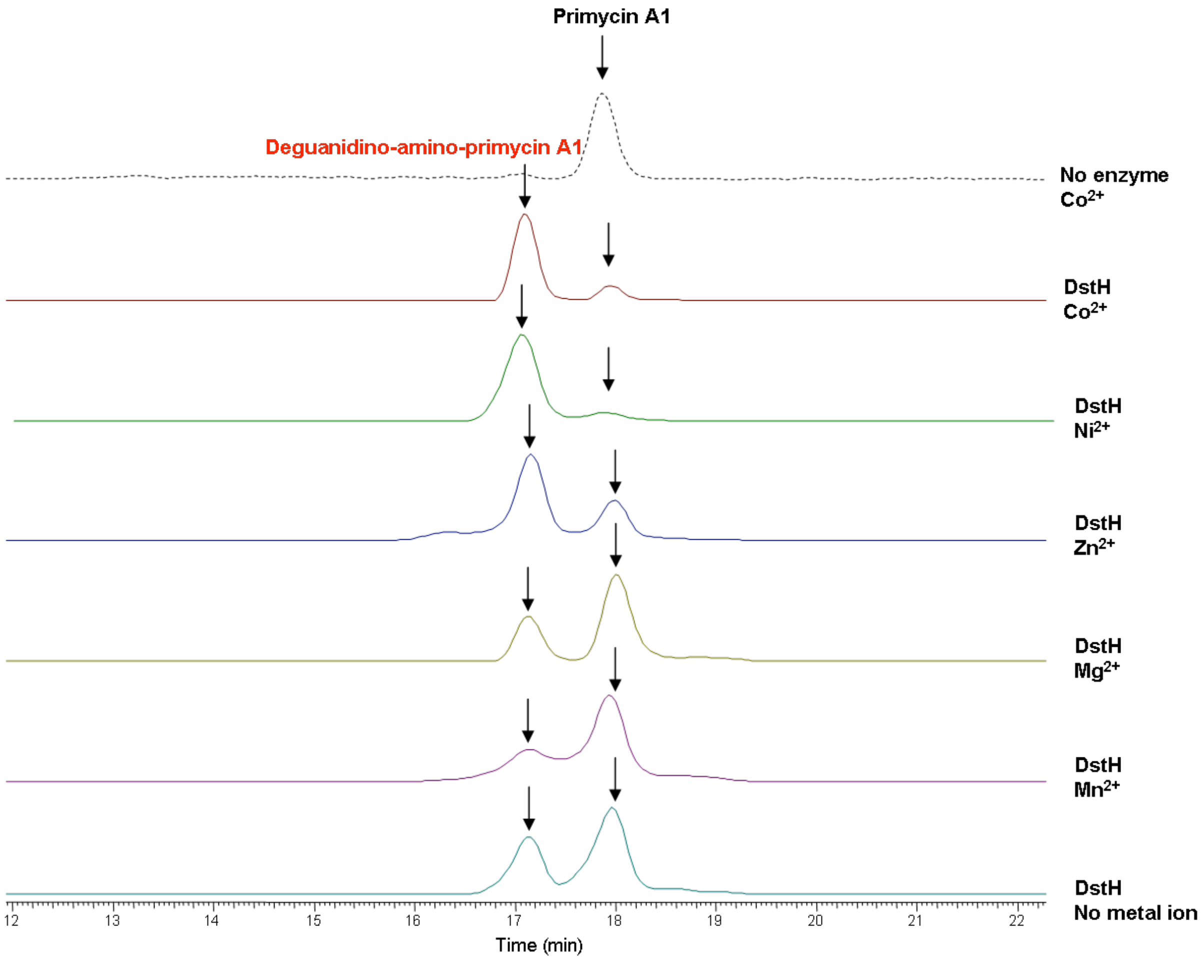

Figure S12. HPLC-ESI-MS total ion current traces of in vitro convertion of primycin A1 catalysed by DstH with various divalent ions. Primycin A1 can be efficiently converted to its amino form under the assay conditions used.

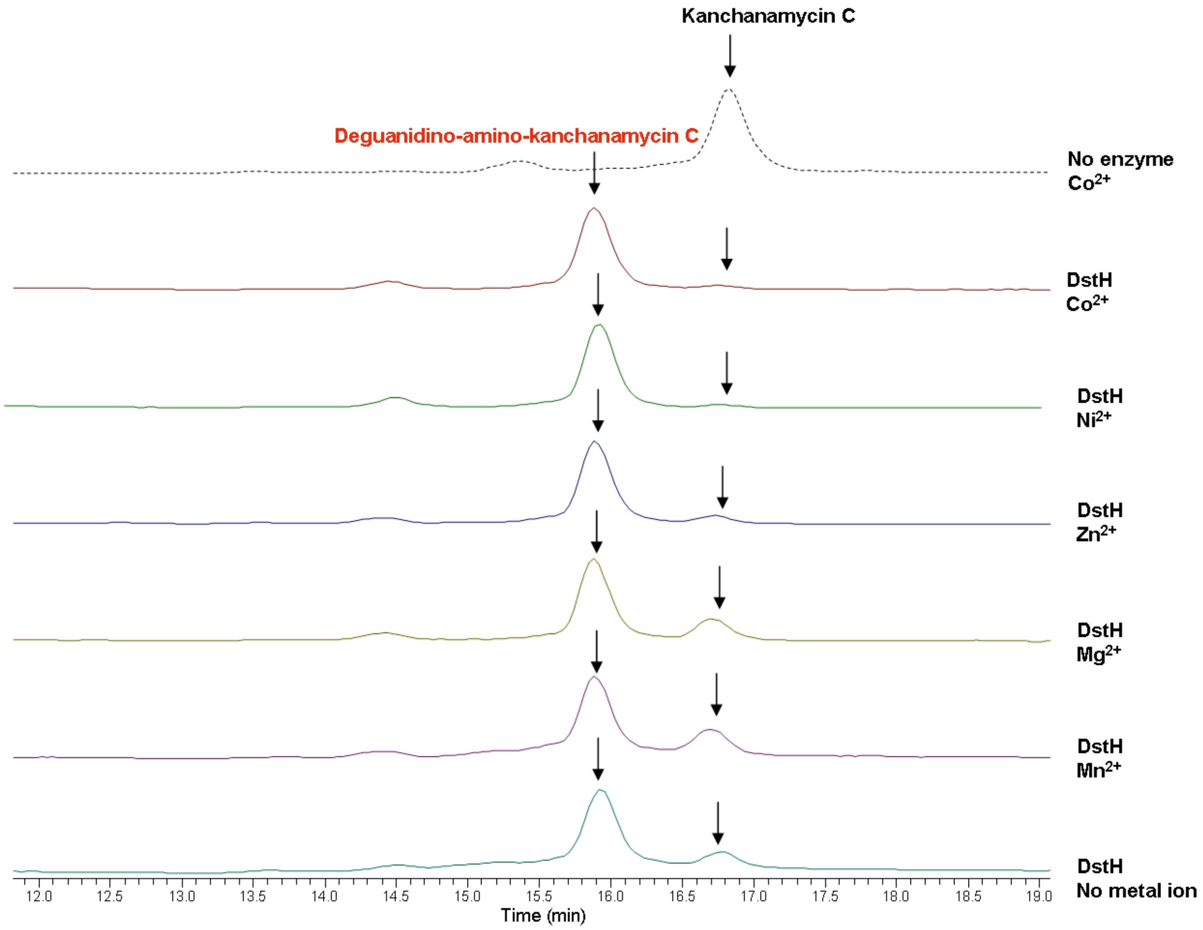

Figure S13. HPLC-ESI-MS total ion current traces of in vitro convertion of kanchanamycin C catalysed by DstH with various divalent ions. Kanchanamycin can be almost completely converted to its amino form under the assay conditions used.

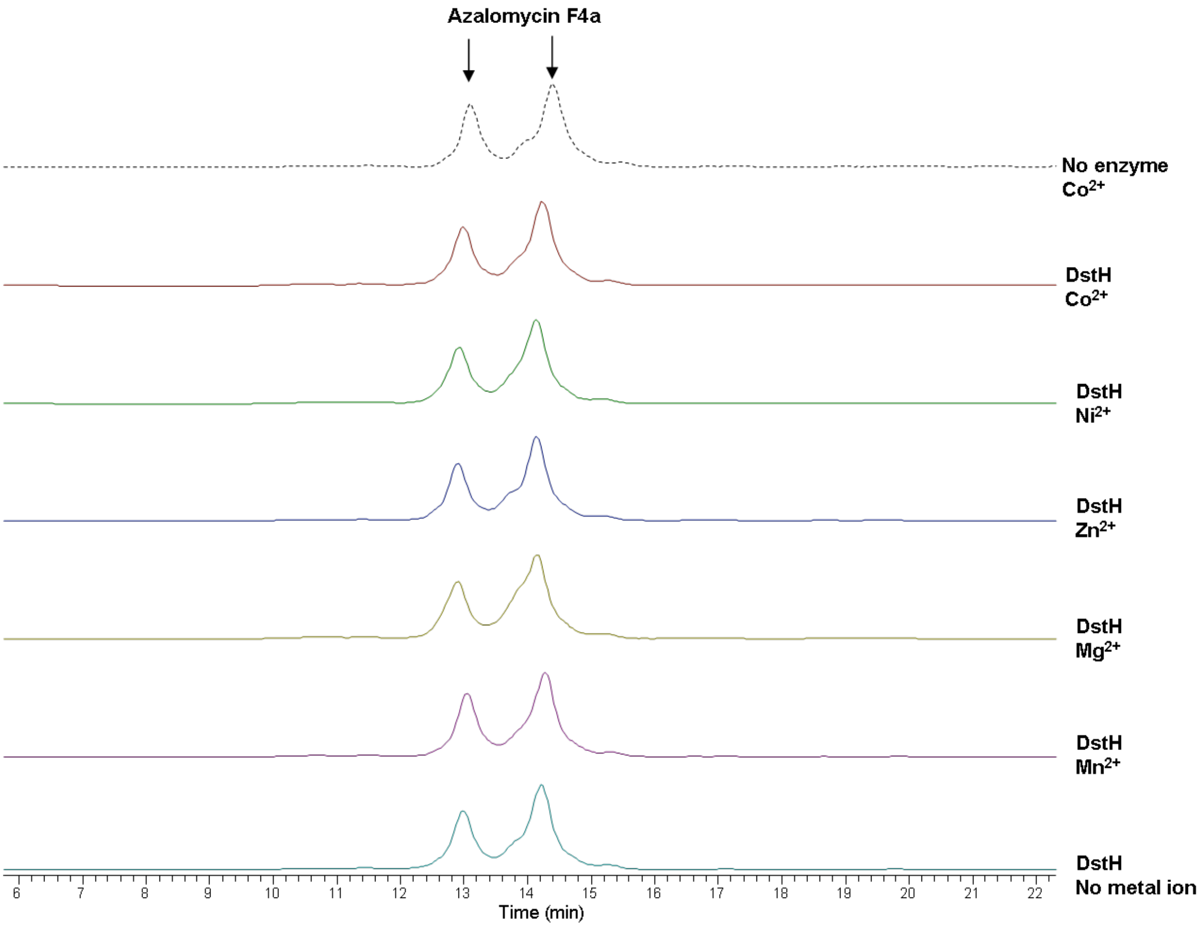

Figure S14. HPLC-ESI-MS total ion current traces of in vitro convertion of azalomycin F4a catalysed by DstH with various divalent ions. Azalomycin F4a can not be converted to its amino form under the assay conditions used. The two peaks are isomers of The two peaks are azalomycin F4a isomers, differing in the attachment site of the malonyl group, either at C23-OH or at C25-OH.[8]

3. Supplementary Tables

Table S1. Bacterial strains used in this study.

Strain	Genotype/Characteristics	Reference
E. coli		
DH10B	F^- mcrA Δ(*mrr*-*hsd*RMS-*mcr*BC), Φ80*lac*ZΔM15, Δ *lac*X74 *rec*A1 *end*A1 *ara*D139 Δ (*ara leu*)7697 *gal*U *gal*K *rpsL nup*G λ–<br>host for general cloning	Invitrogen
BL21(DE3)	F^- ompT $hsdS_B(rB^-, mB^-)$ gal dcm (λDE3 lysogen)<br>host for protein expression	Invitrogen
ET12567 (pUZ8002)	(F^- *dam*-13::Tn9 *dcm*-6 *hsd*M *hsd*R recF143 zjj-202::Tn10 *gal*K2 *gal*T22 *ara*14 *pac*Y1 *xyl*-5 *leu*B6 *thi*-1)<br>Donor strain for conjugation between *E. coli* and *Streptomyces*	[9]

Streptomyces olivaceus Tü4018	Desertomycin A- and kanchanamycin-producing strain	[10]
Streptomyces macronensis	Desertomycin A-producing strain	[11]
Streptomyces spectabilis	Desertomycin A-producing strain	[12]
Saccharomonospora azurea (syn. *S. caesia*)	Primycin-producing strain	[13]
S. violaceusniger DSM4137	Azalomycin-producing strain	[14]

Table S2. Plasmids used in this work.

Plasmid	Genotype/Characteristics	Reference
pYH7	*E.coli-Streptomyces* shuttle vector	[4]
pYH7-dstH	*dstH* gene disruption construct in which a 1635 bp internal fragment of *dstH* was deleted in-frame	this work
pET28a(+)	*E. coli* protein expression vector	Invitrogen
pET28a-dstH	dstH protein expression construct with N-terminal His-tag based on pET28a(+)	this work

Table S3. Oligonucleotide primers used in this work.

Primer	Nucleotide sequence (5' to 3')	Restriction site(s)
primers for protein expression		
dstH-fwd	TTTTCATATGAGCGAGACACCCGAGTCCGA	*Nde*I
dstH-rev	AGCTGAAAGCTT TCACTTGAGCGGGAAGCGCA	*Hin*dIII
primers for dstH gene in-frame deletion		
dstH-up F	TGATCAAGGCGAATACTTCATATGTTCTTCGAGGAGCAGCACCAGGAC	
dstH-up R	GAAGCGGACCTCCTCGCTCATCTCTCTCCTCGGATA	
dstH-dn F	GAGATGAGCGAGGAGGTCCGCTTCCCGCTCAAGTGA	
dstH-dn R	CCGCGCGGTCGATCCCCGCATATGGACGACGTTCAGCACCACGAGGGT	
Primers for PCR screening of mutants		
dstH-CP1	AGACCACCACCAACCTCATCGG	
dstH-CP2	ACGGAGGATGAACGTACCGAAG	

Table S4. Properties of genes within the desertomycin/oasomycin biosynthetic gene cluster of *Streptomyces macronensis* (NRRL B-12566)(A861).

ORF	Product size (aa)	% identity/ similarity	Species	Putative Function	Database entry
dst6241R	181	72/77	*S. clavuligerus*	unknown	EFG10431.1.1
dst6242R	348	46/56	*Stackebrandtia nassauensis*	LuxR regulator	ADD43191.1
dst6243	161	69/79	*S.* sp. C	secreted protein	ZP_05506603.1
dst6244	344	54/68	*Saccharopoly-spora viridis*	esterase	ACU98146.1
dst6245	260	73/83	*S. roseosporus*	3-hydroxy-butyrate de-hydrogenase	EFE73556.1
dst6246	455	84/92	*S.* sp. Mg1	MFS transporter	EDX26472.1
dst6247	667	70/75	*S. clavuligerus*	CdaR regulatory protein	EFG10600.1
dst6248R	650	45/62	*S. scabiei*	ABC transporter	CBG73917.1
dst6249R	394	72/80	*S. hygroscopicus*	alcohol de-hydrogenase	ZP_05518441.1
dst6250	251	48/60	*S. albus*	TetR regulator	ZP_047033876.1
dst6251	285	68/76	*S. avermitilis*	GCN5 related acetyl-transferase	BAC68939.1
dst6252	764	71/80	*S. hygroscopicus*	glucosidase	ZP_05512145.1
dst6253	638	89/94	*S. avermitilis*	ABC transporter	BAC74929.1
dst6254	191	55/72	*Rhodococcus opacus*	TetR regulator	BAH52357.1
dst6255R	278	51/65	*S.* sp. ACTE	4'-phospho-pantetheine transferase	EFB64522.1
dst6256R	416	68/78	*S. avermitilis*	dolichol-P-mannosyl transferase	BAC71004.1

dst6257R	160	—	—	—	—
dst6258	939	40/53	*Salinispora tropica*	LuxR regulator	ABP55203.1
dst6259R	201	42/54	*S. albus*	ABC transporter	EFE832221.1
dst6260	542	*66/77*	*S. hygroscopicus*	arginine oxidase	ZP_05517733.1
dst6261R	287	52/73	*M. aurantiaca*	ABC transporter	EFA33892.1
dst6262R	314	60/73	*S. aizunensis*	ABC transporter	AAX98195.1
dst6263	5249	55/66	*S. aizunensis*	Type I PKS (DstA1)	AAX98184.1
dst6264	3299	57/68	*S. aizunensis*	Type I PKS (DstA2)	AAX98184.1
dst6265	5030	55/66	*S. aizunensis*	Type I PKS (DstA3)	AAX98186.1
dst6266	4874	54/65	*S. aizunensis*	Type I PKS (DstA4)	AAX98184.1
dst6267	5331	55/66	*S. platensis*	Type I PKS (DstA5)	BAH02269.1
dst6268	3465	57/68	*S. platensis*	Type I PKS (DstA6)	BAH02269.1
dst6269	5450	49/63	*Sorangium cellulosum*	Type I PKS (DstA7)	AAA79984.1
dst6270	4403	54/65	*S. aizunensis*	Type I PKS (DstA8)	AAX98191.1
dst6271R	410	72/80	*S. aureofaciens*	peptidase	ABB05108.1
dst6272	483	81/88	*Frankia* sp. EAN1pec	amidase	ABW13525.1
dst6273R	446	46/61	*S.* sp.AA4	glycosyl-transferase	WP_03068347.1
dst6274	478	61/75	*S. aizunensis*	acyl-CoA ligase	AAX98201.1
dst6275	306	52/71	*S. aizunensis*	acyl-transferase	AAX98193.1
dst6276	419	46/62	*Saccharopoly-spora erythraea*	cytochrome P450	WP_03068340.1
dst6277	372	64/77	*Strepto-sporangium*	amidino-hydrolase	ACZ87232.1

			roseum		
dst6278	249	61/77	*S. natalensis*	oleoyl-ACP hydrolase	CAC20922.1
dst6279R	66	—	—	—	—
dst6280	21	—	—	—	—
dst6281	96	—	—	—	—
dst6282	200	78/88	*Stackebrandtia nassauensis*	LuxR regulator	ADD41656.1

Putative functions of the encoded proteins were deduced from analyses with the BlastP program (http://blast.ncbi.nlm.nih.gov/Blast.cgi). The % identity/similarity for the protein in the database with the highest end-to-end similarity is indicated. The sequence has been deposited under the accession number XXXXX.

Table S5. Properties of genes within the desertomycin/oasomycin biosynthetic cluster of *Streptomyces olivaceus* Tü4018 (A828).

ORF	Product size (aa)	% identity/ similarity	Species	Putative function	Database entry
dst6622R	1095	82/87	*S. aurantiacus*	SARP regulatory protein	EPH43652.1
dst6623R	121	48/60	*S. fulvissimus*	SnoaL-like	AGK79080.1
dst6624R	141	37/57	*A. erythraea*	ketosteroid isomerase	KGI826 24.1
dst6625	940	55/63	*S. aurantiacus*	SARP regulatory protein	EPH40088.1
dst6626	179	45/58	*S.* sp. NRRL B-1347	fatty acid binding protein	WP_030681528.1
dst6627	161	73/79	*S. iranensis*	phospho-lipase A2	CDR08492.1
dst6628R	395	51/61	*S. bottropensis*	esterase	EMF55930.1
dst6629	154	—	—	—	—
dst6630	248	99/99	*S.* sp. NRRL B-1347	trypsin	WP_030681536.1
dst6631R	143	100/100	*S.* sp. NRRL B-1347	glyoxalase	WP_030631539.1
dst6632	342	98/99	S. sp. NRRL B-1347	esterase	WP_030631541.1
dst6633	265	99/99	*S.* sp. NRRL B-1347	3-hydroxy-butyrate de-hydrogenase	WP_030631543.1
dst6634	439	99/100	S. sp. NRRL B-1347	MFS transporter	WP_030631546.1
dst6635	647	99/99	*S.* sp. NRRL	CdaR	WP_030631549.1

			B-1347	regulatory protein	
dst6636R	dst661	81/87	*S.* aurantiacus	ABC transporter	EPH39610.1
dst6637R	359	99/99	*S.* sp. NRRL B-1347	oxido-reductase	WP_030631560.1
dst6638	295	97/97	*S.* sp. NRRL B-1347	GCN5 related acetyl-transferase	WP_030631563.1
dst6639R	760	99/99	*S.* sp. NRRL B-1347	glucosidase	WP_030631566.1
dst6640	627	99/100	*S.* sp. NRRL B-1347	ABC transporter	WP_030631569.1
dst6641R	198	99/100	*S.* sp. NRRL B-1347	deaminase/ reductase	WP_030631572.1
dst6642	200	100/100	*S.* sp. NRRL B-1347	TetR regulator	WP_030631573.1
dst6643R	233	58/69	*S.* sp. *CNS654*	4'-phospho-pantetheine transferase	WP_032768805.1
dst6644R	436	99/99	*S.* sp. NRRL B-1347	dolichol-P-mannosyl-transferase	WP_030631577.1
dst6645	916	44/56	*Kutzneria albida*	LuxR regulator	AHH94593.1
dst6646R	224	41/58	C*ellulomonas* sp. HZM	ABC transporter	WP_029291236.1
dst6647	542	99/99	*S.* sp. NRRL B-1347	arginine oxidase	WP_030631582.1
dst6648R	271	96/98	*S.* sp.	ABC transporter	WP_030631563.1
dst6649R	314	99/99	*S.* sp. NRRL B-1347	ABC transporter	WP_030357193.1
dst6650	5091	57/68	*S. sp.* PRh5	Type I PKS (DstA1)	EXU66032.1
dst6651	3247	59/69	*S. violaceusniger*	Type I PKS (DstA2)	AEM87325.1
dst6652	5018	57/68	*S. violaceusniger*	Type I PKS (DstA3)	AEM83813.1

dst6653	4706	50/61	*M. aurantiaca*	Type I PKS (DstA4)	ADL46006.1
dst6654	5232	55/67	*S. sp.* PRh5	Type I PKS (DstA5)	EXU62707.1
dst6655	3399	56/68	*S. violaceusniger*	Type I PKS (DstA6)	AEM83812.1
dst6656	5303	51/62	*S. griseus*	Type I PKS (DstA7)	EGE45820.1
dst6657	4257	56/68	*S. rapamycinicus*	Type I PKS (DstA8)	AGP57754.1
dst6658R	531	48/63	*Saccharomono-spora azurea*	glycosyl transferase	EHK86573.1
dst6659	459	99/99	*S.* sp. NRRL B-1347	acyl-CoA ligase	WP_03068349.1
dst6660	316	98/99	*S.* sp. NRRL B-1347	acyl-transferase	WP_03068347.1
dst6661	446	90/96	*S. aurantiacus*	cytochrome P450	EPH42958.1
dst6662	372	99/99	*S.* sp. NRRL B-1347	amidino-hydrolase	WP_03068343.1
dst6663	248	100/100	*S.* sp. NRRL B-1347	oleoyl-ACP hydrolase	WP_03068340.1
dst6664R	225	—	—	—	—
dst6665R	158	—	—	—	—
dst6666	196	99/100	*S.* sp. NRRL B-1347	LuxR regulator	WP_03068954.1

Putative functions of the encoded proteins were deduced from analyses with the BlastP program (http://blast.ncbi.nlm.nih.gov/Blast.cgi). The % identity/similarity for the protein in the database with the highest end-to-end similarity is indicated. The sequence has been deposited under the accession number XXXXX.

Table S6. Properties of genes within the desertomycin/oasomycin biosynthetic cluster of *Streptomyces spectabilis* NRRL B-2494.

ORF	Product size (aa)	% identity/ similarity	Species	Putative function	Database entry
dst1331R	1095	82/87	*S. aurantiacus*	SARP regulatory protein	EPH43652.1
dst1330R	121	48/60	*S. fulvissimus*	SnoaL-like	AGK79080.1
dst1329R	141	37/57	*A. erythraea*	ketosteroid isomerase	KGI826 24.1

dst1328	180	73/80	*S. fulvissimus*	fatty acid binding protein	AGK81799.1
dst1327	161	68/79	*S. globisporus*	phospho-lipase A2	CDR08492.1
dst1326	274	99/99	*S.* sp. NRRL B-1347	trypsin	WP_030681536.1
dst1325	143	96/97	*S.* sp. NRRL B-1347	glyoxalase	WP_030631539.1
dst1324	340	93/95	S. sp. NRRL B-1347	esterase	WP_030631541.1
dst1323	265	96/97	*S.* sp. NRRL B-1347	3-hydroxy-butyrate de-hydrogenase	WP_030631543.1
dst1322	478	95/97	S. sp. NRRL B-1347	MFS transporter	WP_030631546.1
dst1321	647	95/96	*S.* sp. NRRL B-1347	CdaR regulatory protein	WP_030631549.1
dst1320R	661	81/88	*S. aurantiacus*	ABC transporter	EPH39610.1
dst1319R	352	94/96	*S.* sp. NRRL B-1347	oxido-reductase	WP_030631560.1
dst1318	290	82/89	*S.* sp. NRRL B-1347	GCN5 related acetyl-transferase	WP_030631563.1
dst1317R	758	90/93	*S.* sp. NRRL B-1347	glucosidase	WP_030631566.1
dst1316	627	98/99	*S.* sp. NRRL B-1347	ABC transporter	WP_030631569.1
dst1315R	198	93/96	*S.* sp. NRRL B-1347	deaminase/ reductase	WP_030631572.1
dst1314	200	91/96	*S.* sp. NRRL B-1347	TetR regulator	WP_030631573.1
dst1313R	233	57/68	*S. tsukubaensis*	4'-phospho-pantetheine transferase	EIF93070.1
dst1312R	443	93/96	*S.* sp. NRRL B-1347	dolichol-P-mannosyl-transferase	WP_030631577.1
dst1311	916	39/53	*A. orientalis*	LuxR regulator	ABM47005.1

dst1310R	221	34/52	*Longispora albida*	ABC transporter	WP_018349120.1
dst1309	542	94/98	*S.* sp. NRRL B-1347	arginine oxidase	WP_030631582.1
dst1308R	271	94/98	*S. aurantiacus*	ABC transporter	EPH42743.1
dst1307R	314	95/97	*S.* sp. NRRL B-1347	ABC transporter	WP_030631585.1
dst1306	5067	55/67	*S.violaceusniger*	Type I PKS (DstA1)	AEM83817.1
dst1305	3226	59/70	*S.violaceusniger*	Type I PKS (DstA2)	AEM87325.1
dst1304	4990	57/68	*S.violaceusniger*	Type I PKS (DstA3)	AEM83813.1
dst1303	4717	55/65	*S. zinciresistens*	Type I PKS (DstA4)	EGX61517.1
dst1302	5204	55/67	*S. zinciresistens*	Type I PKS (DstA5)	EGX61515.1
dst1301	3394	55/67	*S.violaceusniger*	Type I PKS (DstA6)	AEM83812.1
dst1300	5315	51/62	*S. griseus*	Type I PKS (DstA7)	EGE45820.1
dst1299	4218	56/67	*S.violaceusniger*	Type I PKS (DstA8)	AEM87318.1
dst1298R	488	46/60	*Amycolatopsis jeuensis*	glycosyl transferase	WP_033289361.1
dst1297	458	96/97	*S.* sp. NRRL B-1347	acyl-CoA ligase	WP_03068349.1
dst1296	316	96/97	*S.* sp. NRRL B-1347	acyl transferase	WP_03068347.1
dst1295	423	89/96	*S. aurantiacus*	cytochrome P450	EPH42958.1
dst1294	372	99/99	*S.* sp. NRRL B-1347	amidino hydrolase	WP_03068343.1
dst1293	248	95/97	*S.* sp. NRRL B-1347	oleoyl-ACP hydrolase	WP_03068340.1
dst1292	225	—	—	—	—
dst1291R	158	—	—	—	—
dst1290	220	99/100	*S.* sp. NRRL B-1347	LuxR regulator	WP_03068954.1

Putative functions of the encoded proteins were deduced from analyses with the BlastP program (http://blast.ncbi.nlm.nih.gov/Blast.cgi). The % identity/similarity for the protein in the database with the highest end-to-end similarity is indicated. The sequence has been deposited under the accession

number XXXXX.

Table S7. Properties of genes within the kanchanamycin biosynthetic cluster of *Streptomyces olivaceus* Tü4018 (A828).

ORF	Product size (aa)	% identity/ similarity	Species	Putative function	Database entry
kch7034	392	99/99	*Streptomyces sp.* NRRL B-1347	MFS transporter	WP_030684614.1
kch7035R	588	98/98	*Streptomyces* sp. NRRL B-1347	chitinase	WP_030684612.1
kch7036	363	92/96	*S. zinciresistens*	cytochrome P450	EGX61513.1
kch7037	68	97/97	*Streptomyces* sp. NRRL B-1347	ferredoxin	WP_030684609.1
kch7038	5181	85/90	*S.* sp. PRh5	Type I PKS (KchA2)	EXU62707.1
kch7039	3378	75/82	*S. zinciresistens*	Type I PKS (KchA3)	WP_007491078.1
kch7040	4721	84/89	*S. rapamycinicus*	Type I PKS (KchA4)	WP_020865963.1
kch7041	8229	88/93	*S.violaceusniger*	Type I PKS (KchA5)	AEM83815.1
kch7042	3170	90/94	*S.violaceusniger*	Type I PKS (KchA6)	WP_014057314.1
kch7043	3460	88/91	*S. zinciresistens*	Type I PKS (KchA7)	WP_007491082.1
kch7044	2111	87/91	*S. iranensis*	Type I PKS (KchA8)	CDR03008.1
kch7045	2296	99/99	*S.* sp. NRRL B-1347	MFS transporter	WP_030683812.1
kch7046	468	99/99	*S.* sp. NRRL B-1347	CoA ligase	WP_030683820.1
kch7047	313	99/99	*S.* sp. NRRL B-1347	acyl transferase	WP_030683822.1
kch7048	26	—	—	—	—
kch7049	559	98/99	*S.* sp. NRRL B-1347	arginine oxidase	WP_030683823.1
kch7050R	241	96/98	*S.* sp. NRRL B-1347	4'-phospho-pantetheine transferase	WP_030683824.1
kch7051R	122	51/52	*S. zinciresistens*	metallo P-esterase	WP_007491096.1

kch7052	257	98/99	*S.* sp. NRRL B-1347	oleoyl-ACP hydrolase	WP_030683829.1
kch7053	154	—	—	—	—
kch7054	91	—	—	—	—
kch7055R	191	99/99	*S.* sp. NRRL B-1347	LuxR regulator	WP_030683833.1
kch7056R	178	51/62	*S.* sp. NRRL S-1824	transposase	WP_030980978.1
kch7057R	469	44/56	*S. iranensis*	transferase	CDR076471.1
kch7058R	507	99/99	*S.* sp. NRRL B-1347	ABC transporter	WP_030683836.1
kch7059R	425	99/99	*S.* sp. NRRL B-1347	regulator	WP_030683838.1
kch7060R	167	86/94	*S. aurantiacus*	putative amidase	WP_016640806.1
kch7061R	155	57/67	*S. fulvoviolaceus*	acetamidase	WP_030604189.1

Putative functions of the encoded proteins were deduced from analyses with the BlastP program (http://blast.ncbi.nlm.nih.gov/Blast.cgi). The % identity/similarity for the protein in the database with the highest end-to-end similarity is indicated. The sequence has been deposited under the accession number XXXXX.

Table S8. Properties of genes within the primycin biosynthetic cluster of S*accharomonospora azurea* DSM 43044 (A821).

**Product size (aa)**	**% identity/ similarity**	**Species**	**Putative function**	**Database entry**
144	68/76	*Saccharomono-spora erythraea*	cytosine deaminase	ZP_06563012.1
477	63/78	*Kribbella flavida*	propionyl-CoA carboxylase-β subunit	ADB30540.1
785	48/64	*Ralstonia pickettii*	penicillin acylase	ACD29296.1
393	49/61	*S. clavuligerus*	membrane protein	EFG05096.1
110	—	—	—	—
262	70/79	*S.* sp. AA4	ABC transporter	CDR08492.1
510	30/48	*Rhodococcus erythropolis*	ABC transporter	BAH02269.1
421	47/64	*Saccharopoly-spora erythraea*	cytochrome P450	CAM05122.1

501	45/60	*S.* sp. AA4	glycosyl-transferase	ZP_05481000.1
338	39/60	*S. aizunensis*	4-guanidino-butanoate: CoA acyl transferase	AAX98193.1
5140	51/64	*S. platensis*	Type I PKS (PriA1)	BAH02269.1
763	30/42	*Saccharopoly-spora viridis*	glucose de-hydrogenase	ACU98636.1
7771	54/65	*S.* sp. FR-008	Type I PKS (PriA2)	AAQ82567.1
5859	55/65	*S. avermitilis*	Type I PKS (PriA3)	NP_821591.1
7102	51/64	*S.* sp. NRRL 30748	Type I PKS (PriA4)	ABC87510.1
6975	53/64	*S. ambofaciens*	Type I PKS (PriA5)	CAJ88175.1
1789	xx/yy	*S. avermitilis*	Type I PKS (PriA6)	NP_821590.1
375	57/75	*Strepto-sporangium roseum*	amidino-hydrolase	ACZ87232.1
67	—	—	—	—
331	65/78	*S. ghanaensis*	ABC transporter	EFE68313.1
269	61/80	*S. lividans*	ABC transporter	EFD68190.1
379	52/68	*S.* sp. AA4	2-component histidine kinase	ZP_05478573.1
208	69/83	*S.* sp. AA4	TetR regulator	ZP_05478572.1
955	45/58	*Thermomono-spora curvata*	LuxR regulator	ACY96337.1
224	—	—	—	—
184	—	—	—	—
216	95/97	*Saccharomono-spora viridis*	response regulator	ACU98248.1
224	91/95	*Saccharomono-spora viridis*	ABC transporter	ACU98247.1

440	84/93	*Saccharomonospora viridis*	signal transduction histidine kinase	ACU98246.1
259	78/84	*Saccharomonospora viridis*	DeoR regulator	ACU98245.1
338	64/74	*Saccharopolyspora erythraea*	galactose-1-P-uridylyl transferase	CAM00105.1
351	84/92	*Saccharomonospora viridis*	trypsin	ACU98243.1
159	91/97	*Saccharomonospora viridis*	molybdo-pterin cofactor synthesis protein	ACU98242.1
148	65/75	*Saccharomonospora viridis*	SAF domain protein	ACU98240.1
56	—	—	—	—
95	75/84	*Saccharomonospora viridis*	5-formylTHF carboligase	ACU98238.1
300	90/94	*Saccharomonospora viridis*	UDP-glucose pyrophosphorylase	ACU98237.1
422	93/95	*Saccharomonospora viridis*	molybdo-pterin biosynthetic protein	ACU98236.1
225	78/84	*Saccharomonospora viridis*		ACU98235.1
290	36/48	*Rhodococcus opacus*	membrane protein	BAH53947.1
471	62/74	*S. aizunensis*	CoA ligase	AAX98201.1
202	67/80	*S. hygroscopicus*	oleoyl-ACP hydrolase	ZP_05520438.1
173	—	—	—	—
943	42/57	*Salinispora tropica*	LuxR regulator	ABP55203.1
173	—	—	—	—
210	54/69	*S. viridochromogenes*	guanidinobutyramide hydrolase	ZP_05530153.1
551	74/82	*S. hygroscopicus*	arginine oxidase	ZP_05517733.1
447	94/97	*Saccharomonospora viridis*	glutamate dehydrogenase	ACU98231.1
613	58/70	*Saccharomonospora viridis*	protein kinase	ACU98230.1
609	60/71	*Saccharomonospora viridis*	protein kinase	ACU98229.1
332	90/94	*Saccharomonospora viridis*	tryptophan tRNA synthetase	ACU98228.1
632	83/90	*Saccharomono-*	acyl-CoA	ACU98226.1

		spora viridis	synthetase	
581	82/88	*Saccharomono-spora viridis*	protein kinase	ACU98225.1
522	84/92	*Saccharomono-spora viridis*	dolichol-P-mannosyl transferase	ACU98224.1

Putative functions of the encoded proteins were deduced from analyses with the BlastP program (http://blast.ncbi.nlm.nih.gov/Blast.cgi). The % identity/similarity for the protein in the database with the highest end-to-end similarity is indicated. The sequence has been deposited under the accession number XXXXX.

Table S9. Properties of genes within the azalomycin biosynthetic cluster of *Streptomyces violaceusniger* DSM4137 (A819).

ORF	Product size (aa)	% identity/ similarity	Species	Putative function	Database entry
azl7488R	1552	88/92	*Streptomyces violaceusniger*	hypothetical protein	WP_014057304.1|
azl7489	151	—	—	—	—
azl7490	248	88/95	*S. iranensis*	cytochrome P450	WP_044567507.1
azl7491	68	97/97	*S. iranensis*	ferredoxin	WP_044567505.1
azl7492	5180	85/90	*S.* sp. PRh5	Type I PKS (AzlA2)	EXU62707.1
azl7493	3376	76/83	*S. zinciresistens*	Type I PKS (AzlA3)	WP_007491078.1
azl7494	4713	84/89	*S. rapamycinicus*	Type I PKS (AzlA4)	WP_020865963.1
azl7495	8259	88/93	*S.violaceusniger*	Type I PKS (AzlA5)	AEM83815.1
azl7496	3166	92/95	*S.violaceusniger*	Type I PKS (AzlA6)	WP_014057314.1
azl7497	3453	90/92	*S. zinciresistens*	Type I PKS (AzlA7)	WP_007491082.1
azl7498	2109	87/91	*Streptomyces* sp. NRRL B-1347	Type I PKS (AzlA8)	WP_037826258.1
azl7499	2308	94/96	*S.* sp. NRRL B-1347	Type I PKS (AzlA1)	WP_030683812.1
azl7500	478	99/99	*Streptomyces* sp. PRh5	CoA ligase	WP_037957079.1
azl7501	309	94/96	*Streptomyces* sp. PRh5	acyl transferase	WP_037957076.1
azl7502	126	94/96	*S.violaceusniger*	HxlR regulator	WP_014057322.1
azl7503R	135	93/97	*S.violaceusniger*	endoribo-	WP_014057323.1

azl7504	262	96/98	*S. iranensis*	nuclease hydrolase	WP_044567478.1
azl7505R	474	91/93	*S. zinciresistens*	hypothetical protein	WP_014057325.1
azl7506R	393	98/99	*S.violaceusniger*	membrane protein	WP_014057326.1
azl7507R	197	77/85	*Streptomyces* sp. AcH 505	TetR regulator	WP_041994482.1
azl7508	298	75/82	*Streptomyces* sp. AcH 505	SDR oxido-reductase	WP_040026840.1
azl7509R	257	90/92	*S. iranensis*	GntR regulator	WP_044567474.1
azl7510	267	93/97	*S. iranensis*	guanidino-butyramide hydrolase	WP_044567472.1
azl7511	478	90/94	*S. rapamycinicus*	amino acid transporter	WP_020865944.1
azl7512R	288	92/96	*S. rapamycinicus*	AraC regulator	WP_020865943.1

Putative functions of the encoded proteins were deduced from analyses with the BlastP program (http://blast.ncbi.nlm.nih.gov/Blast.cgi). The % identity/similarity for the protein in the database with the highest end-to-end similarity is indicated. The sequence has been deposited under the accession number XXXXX.

Table S10. High-resolution MS analysis of desertomycin A and desertomycin B produced from *Streptomyces macronensis*.

Compound name	Observed $[M+H]^+$	Calculated $[M+H]^+$	Error (ppm)	Formula
Desertomycin A (major)	1192.7546	1192.7565	-1.6	$C_{61}H_{110}NO_{21}$
Desertomycin B (minor)	1234.7764	1234.7783	-1.5	$C_{62}H_{112}N_3O_{21}$

Table S11. High-resolution MS analysis of desertomycin B produced from *dstH*-deletion mutant of *Streptomyces macronensis*.

Compound name	Observed $[M+H]^+$	Calculated $[M+H]^+$	Error (ppm)	Formula
Desertomycin B	1234.7777	1234.7783	-0.5	$C_{62}H_{112}N_3O_{21}$

Table S12. High-resolution MS analysis of desertomycin A and kanchanamycins produced from *Streptomyces olivaceus* Tü4018.

Compound name	Observed $[M+H]^+$	Calculated $[M+H]^+$	Error (ppm)	Formula
Desertomycin A	1192.7548	1192.7565	-1.4	$C_{61}H_{110}NO_{21}$

Kanchanamycin-1011 (Deguanidino-amino-kanchanamycin C)	1012.6190	1012.6203	-1.3	$C_{53}H_{90}NO_{17}$
Kanchanamycin-1053 (Kanchanamycin C)	1054.6408	1054.6421	-1.2	$C_{54}H_{92}N_3O_{17}$
Kanchanamycin-925	926.6190	926.6199	-1.0	$C_{50}H_{88}NO_{14}$
Kanchanamycin-967	968.6405	968.6417	-1.2	$C_{51}H_{90}N_3O_{14}$

Table S13. High-resolution MS analysis of primycins produced from S*accharomonospora azurea* DSM 43044.

**Compound name**	**Observed $[M+H]^+$**	**Calculated $[M+H]^+$**	**Error (ppm)**	
Primycin-1035 (Deguanidino-amino-primycin A1)	1036.7134	1036.7142	-0.8	$C_{54}H_{102}NO_{17}$
Primycin-1049	1050.7290	1050.7299	-0.9	$C_{55}H_{104}NO_{17}$
Primycin-1063	1064.7444	1064.7455	-1.0	$C_{56}H_{106}NO_{17}$
Primycin-1077 (Primycin A1)	1078.7354	1078.7360	-0.6	$C_{55}H_{104}N_3O_{17}$
Primycin-1091 (Primycin A2)	1092.7505	1092.7517	-1.1	$C_{56}H_{106}N_3O_{17}$
Primycin-1105 (Primycin A3)	1106.7660	1106.7673	-1.2	$C_{57}H_{108}N_3O_{17}$

Table S14. High-resolution MS analysis of azalomycin F4a produced from *Streptomyces violaceusniger* DSM4137.

**Compound name**	**Observed $[M+H]^+$**	**Calculated $[M+H]^+$**	**Error (ppm)**	**Formula**
Azalomycin F4a	1082.6722	1082.6734	-1.1	$C_{56}H_{96}N_3O_{17}$

4. Supplementary References

[1] J. Sambrook, D. W. Russell, Molecular Cloning: A Laboratory Manual, 3rd ed. Cold Spring Harbor Laboratory Press, New York, **2001**.

[2] T. Kieser, M. Bibb, M. Buttner, K. F. Chater, D. A. Hopwood, Practical *Streptomyces Genetics*. The John Innes Foundation, Norwich, **2001**.

[3] A. Juhász, A. Penzés, Z. A. Péteri, J. P. Pallos, D. Seffer, P. Feiszt, M. P. Pesti, C. Fekete, C. Vágvölgyi, Z. Gazdag, G. Papp, WO2011051741, **2011**.

[4] Y. Sun, F. Hahn, Y. Demydchuk, J. Chettle, M. Tosin, H. Osada, P. F. Leadlay, *Nat. Chem. Biol.* **2010**, *6*, 99-101.

[5] D. G. Gibson, L. Young, R. Y. Chuang, J. C. Venter, C. A. Hutchison III, H. O. Smith, *Nat. Methods* **2009**, *6*, 343-345.

[6] A. T. Keatinge-Clay, *Chem. Biol.* **2007**, *14*, 898-908.

[7] Y. Kobayashi, C.-H. Tan, Y. Kishi, *J. Am. Chem. Soc.* **2001**, *123*, 2076-2078.

[8] G. Yuan, K. Hong, H. Lin, Z. She, J. Li, *Mar. Drugs* **2013**, *11*, 817-829.

[9] D. J. MacNeil, K. M. Gewain, C. L. Ruby, G. Dezeny, P. H. Gibbons, T. MacNeil, *Gene* **1992**, *111*, 61-68.

[10] H. Stephan, C. Kempter, J. W. Metzger, G. Jung, O. Potterat, C. Pfefferle, H.P. Fiedler, *J. Antibiot.* **1996**, *49*, 765 – 769.

[11] L. A. Dolak, F. Reusser, L. Baczynskyj, S. A. Mizsak, B. R. Hannon, T. M. Castle, *J. Antibiot.* **1996**, *36*, 13 – 19.

[12] S. Benallaoua, P. Nguyen Van, M. P. De Meo, J. Coulon, G. Dumenil, R. Bonaly, *Can. J. Microbiol.* **1990**, *36*, 609 – 616.

[13] (a) T. Vályi-Nagy, J. Úri, I. Szilágyi, *Nature* **1954**, *174*, 1105–1106; (b) J. Franka, G. Dékány, I. Pelczer, J. W. ApSimon, *Tetrahedron Lett.* **1987**, *28*, 2759-2762; (c) K. Csepregi, A. Valasek, Á. Pénzes, Z. Tóth, É. Í. Kiss, I. Kerepesi, B. Horváth, I. Nagy, C. Fekete, *J. Bacteriol.* **2012**, *194*, 1263.

[14] H. Hong, T. Fill, P. F. Leadlay, *Angew. Chem. Int. Ed.* **2013**, *52*, 13096 – 13099.
